# Multi-omics landscapes across the gastritis-to-carcinoma sequence: Stage-specific molecular evolution and microenvironmental remodeling

**DOI:** 10.1016/j.isci.2026.116913

**Published:** 2026-08-04

**Authors:** Jiayin Ou, Jiaxin Lin, Yuyang Cao, Yongchao Cui, Jiaqi Liu, Jiansong Fang, Jiayu Li, Jingdong Liu, Hu Qian, Zhiheng Xu

**Affiliations:** 1 Chongqing Hospital of The First Affiliated Hospital of Guangzhou University of Chinese Medicine (Chongqing Beibei Hospital of Traditional Chinese Medicine), Chongqing, China; Science and Technology Innovation Center, Guangzhou University of Chinese Medicine, Guangzhou, Guangdong, China; 2 Science and Technology Innovation Center, Guangzhou University of Chinese Medicine, Guangzhou, Guangdong, China; 3 The Third Clinical Medical College, Guangzhou University of Chinese Medicine, Guangzhou, Guangdong, China; 4 Faculty of Engineering, The University of Sydney, Sydney, NSW, Australia; 5 Guangxi Key Laboratory of Environment and Health Research, Guangxi Medical University, Department of Toxicology, School of Public Health, Guangxi Medical University, Nanning, China; 6 The Second Clinical Medical College, Guangzhou University of Chinese Medicine, Guangzhou, Guangdong, China; 7 Breast cancer department, The First People’s Hospital of Foshan (Foshan Hospital Affiliated to Southern University of Science and Technology), School of Medicine, Southern University of Science and Technology, Shenzhen, Guangdong, China

**Keywords:** omics, chronic gastritis, gastric precancerous lesions

## Abstract

The progression from chronic gastritis to gastric cancer is a complex and multistep process. Early detection of this transition is critical for improving patient survival; however, the underlying molecular mechanisms remain incompletely understood. Recent advances in multi-omics technologies have provided powerful tools for high-resolution characterization of disease-associated molecular alterations. In this review, we summarize studies investigating the gastritis-to-carcinoma sequence using genomics, epigenomics, transcriptomics, proteomics, metabolomics, and microbiome analyses. Particular emphasis is placed on evidence from human cohorts, single-cell sequencing, and spatial transcriptomics, which have helped characterize cellular heterogeneity, immune microenvironment remodeling, and cell-cell interactions. We also discuss the role of the microbiome, particularly *Helicobacter pylori*, in disease progression. Integration of these multi-layered datasets can generate mechanistic hypotheses and candidate biomarkers, but most findings require independent validation before clinical implementation. Overall, multi-omics approaches provide valuable insights into gastric carcinogenesis while highlighting the need for cautious interpretation and translational validation.

## Teaser

Gastroenterology; Hepatology; Precision medicine; Microbiome; Computational bioinformatics; Systems biology

## Introduction

Gastric cancer (GC) remains a major global health challenge, ranking fifth in both incidence and mortality worldwide. In 2022, more than 968,000 new cases were diagnosed, resulting in approximately 660,000 deaths. By 2040, the global burden is projected to reach approximately 1.8 million new cases and 1.3 million deaths, representing increases of 63% and 66%, respectively.^1^^,^^2^ These trends highlight the substantial and growing burden of GC and underscore the urgent need for effective strategies for early detection and prevention. Early prevention and timely intervention have therefore become important public health priorities.

Gastric precancerous lesions (GPL) play a central role in the secondary prevention of GC. Effective prevention, early diagnosis, and timely treatment of GPL may markedly slow or even halt progression to GC. Chronic gastritis (CG) includes chronic non-atrophic gastritis (CNAG) and chronic atrophic gastritis (CAG), which may be accompanied by intestinal metaplasia (IM). CNAG, primarily manifested as chronic superficial gastritis (CSG), corresponds to the inflammatory stage of the Correa cascade and is histologically characterized by superficial mucosal inflammation without glandular atrophy.^3^ CAG is defined by the loss of gastric glands and reduced acid secretion.^4^ IM refers to the adaptive replacement of gastric epithelial cells with intestinal-type epithelium following prolonged inflammation and is widely recognized as a representative precancerous lesion.^5^ Dysplasia develops on this background and is characterized by cellular and structural atypia, with higher grades indicating increased malignant potential.6 Broadly, GPL encompasses CAG, IM, and dysplasia; in a narrower sense, it mainly refers to IM and dysplasia, or dysplasia alone.^6^^,^^7^^,^^8^^,^^9^ Recent studies have also identified another metaplastic form—spasmolytic polypeptide-expressing metaplasia (SPEM)—characterized by TFF2 or MUC6 expression in the basal glands and the emergence of chief cell-like features following parietal cell loss. SPEM is considered a precursor to IM and is therefore included within the spectrum of GPL.^10^ Accordingly, CG and GPL together represent a continuum of pathological stages, including CNAG, CAG, SPEM, IM, and dysplasia.

The global prevalence of CNAG remains high, largely attributable to *Helicobacter pylori (H. pylori)* infection. A systematic review estimated that approximately 44.6% of the global population is infected with *H. pylori*, representing a substantial reservoir of potential CNAG cases.^11^ CAG affects approximately one-third of the global population (33.3%), with marked regional variation; prevalence is highest in Asia (37.4%), followed by Europe (30.1%).^12^ A meta-analysis of longitudinal studies reported an annual progression rate of 0.8%–1.3% for CAG, reflecting its persistent and progressive nature.^13^ The pooled prevalence of IM and dysplasia in Asia reaches 16.2% and continues to increase, particularly in East Asian populations.^14^ *H. pylori* infection represents a major shared risk factor underlying these lesions. In terms of malignant potential, the annual incidence of GC is estimated at 0.1%–0.3% in patients with CAG alone and increases to 0.3%–0.6% when IM is present. The 5-year cumulative cancer risk is approximately 2%–5% for mild to moderate dysplasia and may rise to 15%–25% for severe dysplasia.^15^^,^^16^^,^^17^ Despite these epidemiological observations, the molecular mechanisms underlying CAG and GPL progression remain incompletely understood, highlighting the need for further mechanistic investigation.

**Figure 1:**
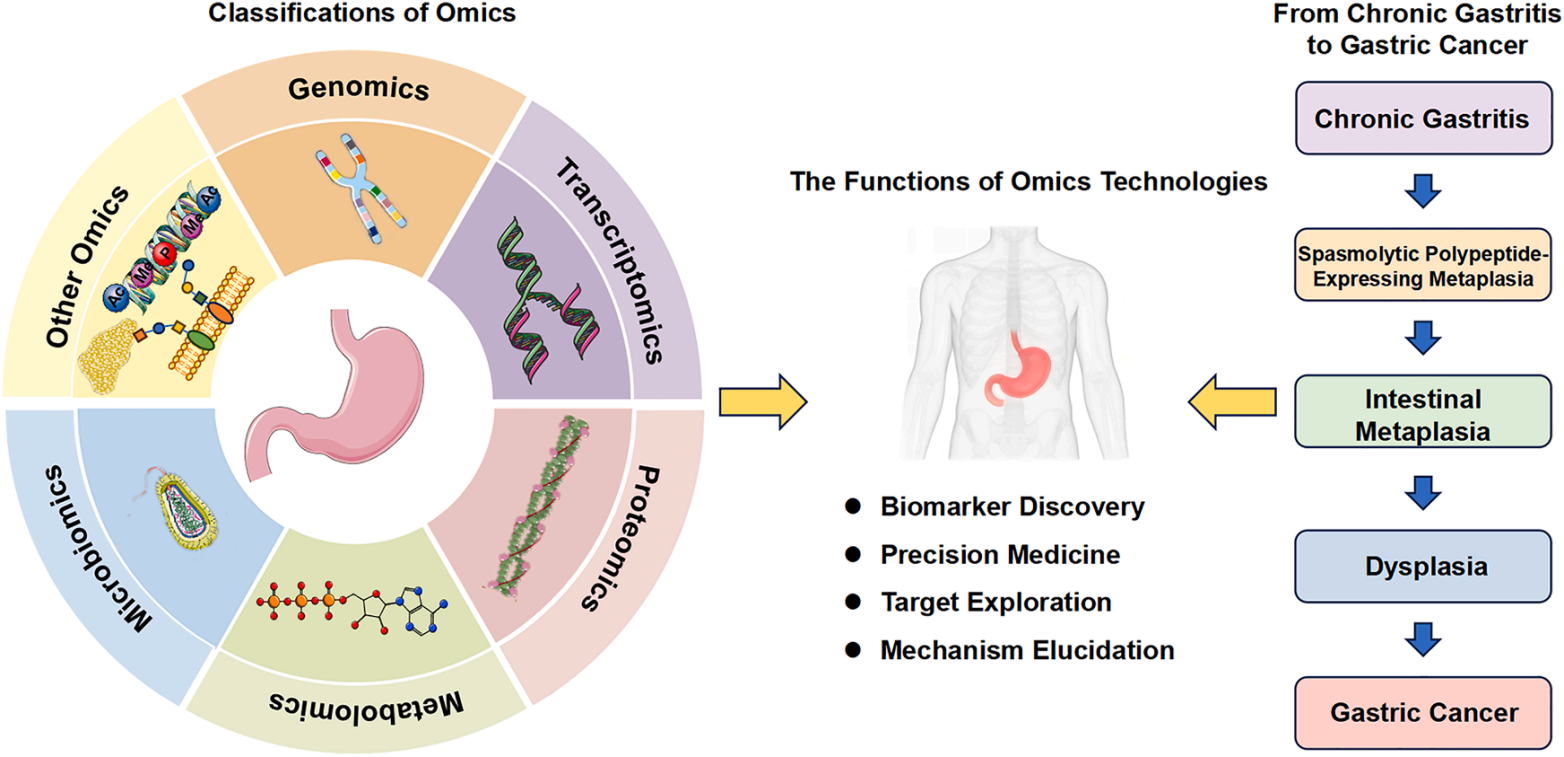
Overview of omics technologies and their conceptual applications across the gastritis-to-carcinoma sequence The figure summarizes major omics layers and their potential contributions to mechanistic investigation, candidate biomarker discovery, and target exploration. These applications should be interpreted as research directions rather than clinically established uses.

Omics technologies—including genomics, transcriptomics, proteomics, metabolomics, microbiomics, and epigenomics—have advanced rapidly in recent years, substantially improving the understanding of disease mechanisms and precision medicine.^18^ These approaches provide systematic frameworks for dissecting complex biological processes and offer insights into disease initiation, progression, diagnosis, and therapeutic intervention. Table 1 summarizes the major omics technologies and their applications in CG and GPL research, highlighting their respective strengths and limitations. Figure 1 illustrates the integration of multi-omics approaches across the gastric carcinogenesis sequence, with emphasis on mechanistic investigation and candidate biomarker discovery rather than immediate clinical deployment. Recent studies published in 2025–2026 have further expanded the application of single-cell and spatial multi-omics technologies in human GPL research, enabling high-resolution characterization of epithelial plasticity, stem-like cell populations, and microenvironmental remodeling across the gastritis-to-carcinoma sequence. Spatial transcriptomic and single-cell multi-omics analyses have identified high-risk precursor signatures enriched in intestinal stem-like programs, delineated epithelial lineage transition drivers, and revealed stromal-immune interactions within metaplastic lesions, thereby providing additional insights into the early evolutionary dynamics of gastric carcinogenesis.^19^^,^^20^^,^^21^

**Table 1:** Overview of omics technologies and their application characteristics in CG and GPL research

Type of Omics	Definition	Advantages in CG/GPL Research	Challenges and Limitations in CG/GPL Research	Research Recommendations and Application Directions
Genomics	The study of the structure, function, variation, and interactions of all genes in an organism.	Reveals genetic susceptibility loci (e.g., genes associated with *H. pylori* susceptibility and CG risk) and elucidates the hereditary basis of disease.	Fails to directly reflect dynamic gene expression and regulation; most identified loci are correlative, with unclear functional mechanisms.	Combine with functional assays (e.g., CRISPR validation) to confirm candidate genes; integrate with transcriptomic and epigenomic data to elucidate gene–environment interactions.
Transcriptomics	Studies the expression levels of all RNA transcripts within a cell at a specific time or condition.	Dynamically reflects gene expression profiles and identifies key signaling pathways (e.g., inflammation, proliferation, apoptosis) in *H. pylori* infection and carcinogenesis.	mRNA abundance does not always correlate with protein function; variability between bulk-tissue and single-cell samples may influence results.	Apply spatial transcriptomics to resolve mucosal heterogeneity; focus on regulatory networks involving non-coding RNAs (lncRNAs, miRNAs).
Proteomics	Large-scale study of protein expression, modification, interactions, and function.	Directly identifies functional effectors and therapeutic targets; elucidates key roles of post-translational modifications (e.g., phosphorylation) in CG–GC progression.	Technically complex; low-abundance regulatory proteins are difficult to detect; tissue heterogeneity remains a major obstacle.	Develop deep-coverage protocols for gastric mucosa; use phosphoproteomics and related methods to characterize signaling dynamics.
Metabolomics	Systematic qualitative and quantitative analysis of all small-molecule metabolites within a biological system.	Closely reflects phenotype and sensitively captures metabolic reprogramming induced by H. pylori infection or acid alterations; a source of candidate biomarkers requiring validation.	Wide dynamic range and structural diversity of metabolites; compound annotation and pathway databases remain incomplete.	Integrate with microbiomics to elucidate host–microbiota co-metabolism in CG; apply to dynamic evaluation of intervention responses.
Microbiomics	Investigates the composition, function, and host interactions of microbial communities (e.g., gastric and intestinal microbiota).	Reveals interactions between *H. pylori* and non-*H. pylori* species, highlighting the central role of gastric microecological imbalance in CAG pathogenesis.	Causality between microbiota alterations and disease remains unclear; large inter-individual variability; strongly influenced by diet and medication.	Conduct longitudinal cohort studies; combine metagenomic/metatranscriptomic approaches to assess microbial activity; employ fecal transplantation to validate causality.
Epigenomics	Studies heritable gene regulation mechanisms that do not involve DNA sequence changes (e.g., DNA methylation, histone modification).	Links genetic and environmental factors (e.g., *H. pylori* infection, diet); hypermethylation-mediated gene silencing serves as a key driver of precancerous lesions.	Highly cell- and tissue-specific; epigenetic alterations are often subtle and complex; causality is difficult to establish.	Apply single-cell epigenomics to characterize cell subtype–specific changes; explore the therapeutic potential of epigenetic-targeting drugs.
Lipidomics	Systematic analysis of lipid molecular structures, functions, and metabolic dynamics within biological systems.	Reveals lipid mediators involved in gastric mucosal barrier integrity and inflammation (e.g., prostaglandins) and their roles in CG pathology.	Structural complexity and isomeric diversity of lipids complicate analysis; broad dynamic detection range required.	Focus on sphingolipid and arachidonic acid metabolism related to inflammation and carcinogenesis; integrate with imaging for spatial localization.
Glycomics	Comprehensive study of glycan structures, biosynthesis, and biological functions.	Investigates glycosylation of mucosal barrier proteins (e.g., mucins MUC5AC and MUC6) and its dual role in mucosal protection and carcinogenesis prevention.	Glycan structures are highly complex and branched; analytical standardization remains challenging.	Develop advanced mass spectrometry–based glycan structural characterization methods; explore glycomics as a noninvasive diagnostic tool (e.g., serum glycome biomarkers).
Microbiomics	Investigates the composition, function, and host interactions of microbial communities (e.g., gastric and intestinal microbiota).	Reveals interactions between *H. pylori* and non-*H. pylori* species, highlighting the central role of gastric microecological imbalance in CAG pathogenesis.	Causality between microbiota alterations and disease remains unclear; large inter-individual variability; strongly influenced by diet and medication.	Conduct longitudinal cohort studies; combine metagenomic/metatranscriptomic approaches to assess microbial activity; employ fecal transplantation to validate causality.

Abbreviations: GC, gastric cancer; GPL, gastric precancerous lesions.

Genomics investigates the structure, function, evolution, and regulation of the genome through DNA sequencing and analysis of sequence variation.^22^ Transcriptomics analyzes the complete set of RNA transcripts under specific conditions, providing insights into gene expression regulation and cellular plasticity relevant to mechanistic studies and diagnostics.^23^ Proteomics explores the composition, function, and interactions of proteins in cells, tissues, or organisms, typically using mass spectrometry and two-dimensional electrophoresis to characterize protein expression, modifications, and interactions.^24^ Metabolomics examines global alterations and functional dynamics of small metabolites influenced by environmental and physiological factors, integrating analytical techniques with bioinformatics to identify metabolic patterns and pathways.^25^ Microbiomics investigates the composition and functional dynamics of microbial communities and their interactions with the host and environment.^26^ Epigenomics studies heritable regulation of gene expression independent of DNA sequence alterations, including DNA methylation, histone modifications, and chromatin remodeling, thereby providing insights into disease-associated regulatory mechanisms.^27^

Recent advances in multi-omics integration have shifted the understanding of the gastritis-to-carcinoma sequence from isolated molecular alterations toward coordinated cross-layer regulatory processes.^28^ Genomic and epigenetic alterations are increasingly recognized as drivers of downstream transcriptional, proteomic, and metabolic reprogramming. Emerging evidence also highlights interactions among microbial dysbiosis, inflammatory signaling, and metabolic remodeling in shaping the gastric microenvironment. Furthermore, single-cell multi-omics approaches have revealed substantial cellular heterogeneity and lineage plasticity during key transitional stages such as SPEM and IM. Collectively, these findings support a systems-level framework for understanding gastric carcinogenesis and provide a conceptual basis for integrative multi-omics studies in this field.

This review summarizes current applications and emerging trends of both individual and integrated omics approaches in CNAG, CAG, SPEM, IM, and dysplasia, with particular emphasis on recent advances in single-cell and spatial multi-omics, microbiome-associated mechanisms, and integrative frameworks for biomarker discovery, mechanistic investigation, and precision prevention across the gastritis-to-carcinoma sequence.

This narrative review was based on literature retrieved from PubMed, Web of Science, and Scopus databases. Publications from the past decade focusing on CG, GPL, and omics-related research were preferentially included. Particular attention was given to recent advances in human single-cell and spatial multi-omics studies, microbiome-associated mechanisms, and integrative computational approaches. Relevant references cited in key articles were also manually screened to ensure balanced coverage of the field.

## Chronic non-atrophic gastritis

CNAG represents the early stage of chronic gastric mucosal inflammation and is characterized by the absence of glandular atrophy. It primarily includes CSG, in which inflammation is confined to the superficial layer of the gastric mucosa (the upper one-third of the lamina propria) and is accompanied by infiltration of lymphocytes and plasma cells without glandular loss.^29^ *H. pylori* infection is the most common etiological factor. Within the Correa cascade, CNAG represents the initial stage of gastric carcinogenesis. Persistent *H. pylori* infection sustains chronic inflammation, and a subset of cases may progress to CAG, IM, dysplasia, and eventually GC.^30^ Therefore, timely eradication of *H. pylori* and early intervention in CNAG are important for interrupting disease progression. The following section summarizes omics-based studies in CNAG and their implications for early intervention and potential therapeutic strategies targeting GPL. CNAG is mainly characterized by chronic inflammatory activation and early microbial alterations. Table 2 summarizes omics-based findings in CNAG, highlighting inflammatory activation, *H. pylori*-associated microbial alterations, and early changes in immune-related signaling pathways.

**Table 2:** Applications and findings of omics technologies in the research of CNAG and CSG

Omics Technology	Sample Information	Specific Techniques	Major Findings	Reference
Transcriptomics	Human gastric mucosa	scRNA-seq	The ITGB2–CXCL1–CXCR2 axis recruits MDSCs to remodel the microenvironment; ITGB2 expression is markedly higher in CSG than in CNAG and positively correlated with CXCL1 and overexpression of ITGB2 promotes CXCL1 secretion, accelerating inflammation-to-cancer transformation.	Shang et al.^31^
	Mongolian gerbil gastric mucosa	scRNA-seq	During H. pylori-associated gastric carcinogenesis, Cx43 decreases and GATA-3 increases at the CNAG stage; GATA-3 may contribute to Cx43 downregulation and impaired gap junctions during early disease progression.	Liu et al.^32^
Proteomics	Human serum	LC-MS/MS	Serum ITIH4 is significantly reduced in CSG and may serve as an early warning biomarker for GC.	Sun et al.^33^
	Human gastric mucosa	LC-MS/MS	ITGB2 is markedly upregulated at the CNAG stage and increases progressively with lesion severity; through the CXCL1–CXCR2 axis, it is associated with inflammatory activation and may represent a candidate biomarker for early lesion progression.	Shang et al.^31^
	Human serum	SELDI-TOF-MS	Expression of fibrinogen α-chain, Apo A-II, and Apo C-I in CSG approximates that in healthy subjects, whereas these proteins are markedly altered in GC, suggesting their potential as GC progression markers.	Liu et al.^34^
	Human gastric mucosa	LC-MS/MS	CMTM4 decreases progressively from CSG to GC; its overexpression suppresses proliferation and invasion via STAT1, serving as a potential early biomarker and therapeutic target.	Han et al.^35^
Metabolomics	Rat gastric mucosa and serum	UHPLC-Orbitrap-MS/MS	Elevated phospholipid and amino acid metabolites and reduced betaine levels were observed in CSG serum, indicating disordered lipid and energy metabolism. *Jinhuawan* reversed these abnormalities and modulated the NO pathway.	Xiao et al.^36^
	Rat gastric mucosa and feces	GC-TOF-MS	Serum lactate and pyruvate levels are significantly increased in CNAG, indicating that glycolytic reprogramming is an early metabolic hallmark of GPL.	Yu et al.^37^
	Human urine	ˆ1H-NMR	In both *Pi-deficiency* and *Pi-Wei damp-heat* CSG syndromes, metabolites such as hippuric acid and lactate differ markedly, suggesting that energy metabolism and gut dysbiosis underlie TCM syndrome differentiation.	Wang et al.^38^
	Human serum	UHPLC-QE-MS	*Evodia decoction* rebalanced six lysophosphatidylcholines and metabolites in the tryptophan–kynurenine pathway in CNAG patients, alleviating inflammation-induced oxidative stress and energy dysregulation.	Hu et al.^39^
Microbiomics	Human gastric mucosa	16S rRNA-seq	CNAG gastric microbiota is enriched in *Streptococcus* and *Rothia*, characterized by activated short-chain fatty acid (SCFA) synthesis pathways, suggesting microbial contribution to mucosal repair and maintenance of superficial gastritis.	Yao et al.^40^
	Human and mouse gastric mucosa	16S rRNA-seq	Transplantation of CNAG-derived microbiota into germ-free mice reproduced superficial inflammation and epithelial hyperplasia in recipient gastric tissue.	Kwon et al.^41^
	Human gastric mucosa	16S rRNA-seq	CSG gastric mucosa shows enrichment of *Lactobacillus*, *Prevotella*, and *Rothia* with enhanced SCFA production, suggesting a protective role in maintaining non-atrophic mucosal homeostasis.	Li et al.^14^^,^^15^^,^^42^^,^^43^^,^^44^
Multi-omics	Human gastric mucosa, serum, and urine	16S rRNA-seq; UHPLC-QE-MS; scRNA-seq	*Jinhuawan* suppresses *Helicobacter pylori*, restores *Lactobacillus*, rebalances the kynurenine–tryptophan pathway and IL-17 signaling, and reverses the dysregulation of the microbiota–metabolite–immune network in CSG.	Xiao et al.^36^

Abbreviations: CNAG, chronic non-atrophic gastritis; CSG, chronic superficial gastritis; scRNA-seq, single-cell RNA sequencing; LC-MS/MS, liquid chromatography-tandem mass spectrometry; SELDI-TOF-MS, surface-enhanced laser desorption/ionization time-of-flight mass spectrometry; UHPLC-Orbitrap-MS/MS, ultra-high-performance liquid chromatography-orbitrap tandem mass spectrometry; GC-TOF-MS, gas chromatography-time-of-flight mass spectrometry; ˆ1H-NMR, proton nuclear magnetic resonance spectroscopy; UHPLC-QE-MS, ultra-high-performance liquid chromatography-Q Exactive mass spectrometry; 16S rRNA-seq, 16S ribosomal RNA sequencing.

### Transcriptomics

Transcriptome sequencing combined with bioinformatic analyses demonstrated that ITGB2 promotes the recruitment of myeloid-derived suppressor cells through activation of the CXCL1-CXCR2 axis, thereby contributing to immune microenvironment remodeling. Compared with CNAG tissues, ITGB2 expression was significantly increased in CSG and positively correlated with CXCL1 levels. Functional assays further showed that ITGB2 overexpression enhanced CXCL1 secretion from gastric epithelial cells, potentially facilitating inflammation-associated disease progression.^31^ During *H. pylori*-associated gastric carcinogenesis, Connexin43 expression decreased whereas GATA-3 expression increased from normal gastric mucosa to the CNAG stage. These findings suggest that inflammatory upregulation of GATA-3 may contribute to Connexin43 downregulation and disruption of gap junctions during early disease progression.^32^

### Proteomics

Proteomic analysis using liquid chromatography-tandem mass spectrometry (LC-MS/MS) revealed that serum inter-α-trypsin inhibitor heavy chain 4 (ITIH4) was significantly downregulated during the CSG stage, suggesting its potential as an early biomarker associated with GC risk.^33^ Proteomic profiling further showed that ITGB2 expression was elevated at the CNAG stage and continued to increase during progression from CG to low-grade intraepithelial neoplasia and early GC. Through activation of the CXCL1–CXCR2 axis and promotion of inflammatory responses, ITGB2 may represent a candidate biomarker associated with early lesion progression.^31^

Surface-enhanced laser desorption/ionization time-of-flight mass spectrometry (SELDI-TOF-MS)-based serum proteomic profiling demonstrated that the expression levels of fibrinogen α-chain, Apo A-II, and Apo C-I in patients with CSG were comparable to those in healthy controls but markedly altered in GC. These findings suggest that later alterations in these proteins may reflect disease progression.^34^ Proteomic analysis further demonstrated that CMTM4 expression was higher in CSG mucosa than in GC tissues and gradually decreased during disease progression. Functional studies indicated that CMTM4 overexpression inhibited cell proliferation and invasion through the STAT1 signaling pathway, supporting its potential relevance in gastric carcinogenesis.^35^

### Metabolomics

Metabolomic studies have revealed disturbances in lipid and energy metabolism during CSG, including altered phospholipid and amino acid metabolite profiles.^36^ More directly, non-targeted metabolomic analysis using gas chromatography-time-of-flight mass spectrometry (GC-TOF-MS) showed elevated levels of glycolytic intermediates, including lactate and pyruvate, in the serum of patients with CNAG, suggesting that glucose metabolic reprogramming may represent an early metabolic feature associated with GPL.^37^

Non-targeted urinary metabolomics based on proton nuclear magnetic resonance (ˆ1H-NMR) identified significant differences in metabolites, including hippuric acid and lactic acid, among clinically defined CSG subgroups, suggesting heterogeneity in energy metabolism and microbiota-associated metabolic profiles.^38^ A UPLC-QTOF-MS plasma metabolomics study in patients with CNAG further implicated lysophosphatidylcholine species and the tryptophan-kynurenine pathway in inflammation-associated metabolic imbalance.^39^ However, most currently identified metabolic alterations remain associative, and their mechanistic roles in disease progression require further validation.

### Microbiomics

16S ribosomal RNA sequencing (16S rRNA-seq) in *H. pylori*-negative individuals revealed that the gastric microbiota in CNAG was characterized by the enrichment of Streptococcus and Rothia genera together with the activation of short-chain fatty acid synthesis pathways, suggesting a potential association with maintenance of superficial inflammatory states and mucosal repair.^40^ These findings support the relevance of early microbial alterations in CNAG, but functional studies are still needed to define causal microbe-host interactions.

16S rRNA-seq analysis of gastric mucosa from patients with CNAG, followed by the transplantation of human-derived microbiota into germ-free mice, reproduced superficial inflammation and epithelial hyperplasia, indicating that CNAG-associated microbiota may contribute to disease-related phenotypes.^41^ The gastric mucosal microbiota in CSG showed enrichment of Lactobacillus, Prevotella, and Rothia species, together with active short-chain fatty acid synthesis, suggesting a potential role for these bacteria in maintaining mucosal barrier function.^45^

### Multi-omics integration

Multi-omics integration combines data from genomics, transcriptomics, proteomics, and metabolomics to provide a more comprehensive understanding of biological functions and physiological mechanisms. By overcoming limitations associated with single-omics analyses, this integrative strategy facilitates the identification of cross-layer regulatory relationships and supports construction of molecular networks linking upstream perturbations with downstream functional alterations.

Integrative analyses combining 16S rRNA microbiome profiling and UPLC-QTOF-MS-based metabolomics in serum and gastric mucosa demonstrated that microbial dysbiosis and metabolic alterations were already evident at the CNAG stage. Reduced Lactobacillus abundance and enrichment of Streptococcus were associated with increased phosphatidylcholine levels and decreased bile acids, suggesting that microbiota-associated metabolic shifts may influence the gastric microenvironment.^46^ Collectively, these findings indicate that disruption of microbe-metabolite interactions may contribute to early disease progression, although the underlying causal mechanisms remain to be further clarified.

## Chronic atrophic gastritis

CAG is a common gastric disorder affecting approximately 20% of the global population. The disease not only leads to digestive dysfunction and impaired absorption of iron, folate, and vitamin B12, but is also recognized as a critical precancerous stage in GC.^42^ Epidemiologically, CAG is closely associated with *H. pylori* infection and may present as either diffuse or multifocal atrophy depending on the population. Diagnosis primarily relies on high-definition endoscopy combined with chromoendoscopy or narrow-band imaging, together with multiple biopsies. The Operative Link on Gastritis Assessment (OLGA) and Operative Link on Gastric Intestinal Metaplasia Assessment (OLGIM) staging systems are commonly used to identify high-risk patients. Eradication of *H. pylori* can alleviate or partially reverse gastric mucosal atrophy, although its effects on IM remain limited. Nevertheless, eradication therapy is recommended to delay disease progression. High-risk patients are generally advised to undergo endoscopic surveillance every 1–3 years, whereas longer surveillance intervals may be appropriate for individuals with mild to moderate lesions.^4^^,^^47^ The integration of advanced omics technologies has provided new insights into the pathogenesis of CAG and potential molecular targets associated with disease progression. CAG is characterized by glandular atrophy accompanied by immune and metabolic alterations. Table 3 summarizes representative omics findings in CAG, highlighting immune dysregulation, epithelial atrophy, and metabolic alterations, including changes in lipid metabolism and mitochondrial function.

**Table 3:** Applications and findings of omics technologies in the research of CAG

Omics Technology	Sample Information	Specific Technique	Main Findings	Reference
Genomics	Human blood	GWAS	Identified three novel susceptibility loci (including rs12757669 in 1q32), implicating *C1orf106* and related genes as CAG risk factors.	Han et al.^48^
	Human blood	SNP array	IL-1B-511 C/T, TNF-α-308 G/A, and IL-10-819 C/T polymorphisms significantly increase CAG risk, suggesting that pro-inflammatory gene variants are associated with mucosal atrophy.	Yan et al.^49^
	Human blood	GWAS	Sex-specific differences observed in AAG: females carrying the HLA-DRB1∗06 allele show higher frequency, suggesting immune modulation as a mechanism for increased risk.	Lahner et al.^50^
	Human blood	GWAS; SNP analysis	Epigenetic interactions between *ERCC6* and *ERCC8* SNPs were identified; the *ERCC6* rs1917799 and *ERCC8* rs158572 combination correlates with reduced CAG risk.	Jing et al.^51^
	Human blood	GWAS	ERCC8 SNPs (rs158572, rs158916) interact significantly with H. pylori infection, smoking, and alcohol consumption, potentially modifying CAG risk.	Jing et al.^52^
Transcriptomics	Human gastric mucosa	scRNA-seq	Among 143 CAG-related pathogenic genes, low expression of *GADD45B* emerged as a core oncogenic factor, associated with poor prognosis and altered immune microenvironment.	W. Xu et al.^53^
	Human gastric mucosa	DNBSEQ-G99 RNA-seq	Constructed a lncRNA/circRNA–miRNA–mRNA regulatory network; identified differentially expressed ncRNAs and mRNAs potentially regulating signaling pathways in CAG.	Pan et al.^54^
	Human gastric mucosa	DNBSEQ-G99 RNA-seq; scRNA-seq	H. pylori infection upregulates HKDC1, which increases with CAG severity; is associated with activation of the TGF-β/Smad2/EMT pathway and may contribute to gastric injury and precancerous progression.	Giannakis et al.^55^
	Human gastric mucosa	scRNA-seq	A 22,511-cell atlas identified a high-metabolic epithelial subpopulation (cluster_4) in EGC. ERO1LB was associated with enhanced glutamine metabolism, tumor proliferation, migration, and poor prognosis, suggesting potential relevance to disease progression.	Ni et al.^56^
Proteomics	Human gastric mucosa	LC-MS/MS	*ITGB2* is significantly upregulated in CAG mucosa and activates NF-κB via the CXCL1–CXCR2 axis, promoting inflammatory progression.	Shang et al.^31^
	Mouse gastric mucosa	LC-MS/MS	Mitochondrial protein *Ndufs8* is markedly downregulated in CAG mice; hUC-MSC transplantation restores *Ndufs8*-mediated mitophagy and alleviates CAG pathology.	Rui et al.^57^
	*H. pylori* strains	MALDI-TOF/MS	Comparative proteomics revealed *peroxiredoxin* and *HSP70* as potential CAG biomarkers.	Bernardini et al.^58^
	Human gastric mucosa	2D-DIGE; 2D-DIGE; LC-MS/MS	Identified 53 differential proteins in the gastric corpus involving TCA cycle dysfunction, some shared with GC, suggesting AAG progression toward malignancy.	Repetto et al.^59^
	Human gastric mucosa	2D-DIGE; MALDI-TOF/TOF MS	Identified 15 differential *H. pylori* proteins potentially serving as AAG biomarkers.	Lahner et al.^60^
Metabolomics	Human serum	ˆ1H-NMR	CAG-associated metabolites are closely linked to energy metabolism disruption, inflammation, immune dysfunction, and oxidative stress, providing novel therapeutic targets.	Cui et al.^61^
	Human urine	ˆ1H-NMR	CAG-related metabolites are associated with mitochondrial function, TCA cycle activity, and redox imbalance.	Liu et al.^62^
	Tongue coating scrapings	GC-TOF-MS; UHPLC-QE-MS	Sphingosine-1-phosphate levels correlate positively with CAG and IM progression, serving as diagnostic markers.	You, Lu et al.^63^
	Tongue coating scrapings	GC-TOF-MS; UHPLC-QE-MS	Identified lipid-derived metabolites such as α-Carboxy-δ-decalactone as potential biomarkers in tongue-coating samples.	You, Zhang et al.^64^
	Rat urine	ˆ1H-NMR; UHPLC-QE-MS; UHPLC-QE-MS	Identified 15 CAG-related metabolites; *Huangqi Jianzhong Decoction* reverses metabolic disorders to exert therapeutic effects.	Liu et al.^65^
	Rat serum	UPLC-Q-TOF/MS	Dehydroevodiamine ameliorates NMNG-induced CAG via suppression of HIF-1α–mediated VEGF signaling.	Wen et al.^66^
	Rat serum	ˆ1H-NMR	The aqueous extract of *Gastrodia elata* regulates energy and amino acid metabolism to exert anti-inflammatory and antioxidant effects, improving CAG pathology.	Xu et al.^67^
Microbiomics	Human feces	16S rRNA-seq	Odd-chain fatty acids significantly reduced in CAG and correlate with decreased α-diversity and altered abundance of *Blautia* and *Faecalibacterium*.	Gai et al.^68^
	Human gastric mucosa	16S rRNA-seq	AAG may disrupt gastric acidity, eliminating sex-related microbial diversity and aggravating dysbiosis.	Pivetta et al.^69^
	Human feces	16S rRNA-seq	*H. pylori*-infected CAG patients show altered *Lactobacillus* abundance, potentially exacerbating disease progression.	Iino et al.^70^
	Rat feces and intestinal contents	16S rRNA-seq	*Hua Zhuo Jie Du Fang* modulates gut microbiota and its metabolites to improve CAG pathology.	Liu et al.^25^
	Rat feces and intestinal contents	16S rRNA-seq	*Cinnamomi Cortex* modulates gut microbial composition and primary bile acid metabolism to alleviate CAG.	Zhou et al.^71^
	Rat gastric mucosa and feces	16S rRNA-seq	Mesenchymal stem cells suppress inflammation, promote gastric stem cell regeneration, and restore microbial balance, preventing GPL progression.	Park et al.^72^
Lipidomics	Rat gastric mucosa and serum	Targeted LC-MS/MS lipidomics	Combined *Panax notoginseng* saponins and aspirin modulate arachidonic acid metabolism, enhancing antiplatelet effects while reducing gastric injury.	Wang et al.^73^
Glycomics	Human gastric mucosa	LC-MS/MS-based glycomics; Lectin microarray-based glycomics	CAG features reduced core type 1/2 O-glycans and elevated sialyl-Tn antigen; aberrant glycosylation activates EGFR/MAPK signaling, driving early tumorigenesis.	Arai et al.^74^
	Human saliva	Lectin microarray-based glycomics; MALDI-TOF-MS-based glycomics; LC-MS/MS-based glycomics	Elevated fucosylation, α2,6-sialylation, and bisecting GlcNAc in CAG saliva suggest diagnostic glycan biomarkers for GPL/CAG detection.	Shu et al.^75^
Epigenomics	Human gastric mucosa	Whole-genome DNA methylation array; Pyrosequencing	Hypermethylation of cancer risk genes reflects overall gastric mucosal methylation load and correlates with CAG severity.	Irie et al.^76^
	Human blood	Illumina 450K methylation array	*H. pylori* (especially CagA^+^ strain) infection and CAG accelerate peripheral blood DNA methylation age, contributing to epigenetic aging.	Wizenty et al.^77^
	Human gastric mucosa	Pyrosequencing	*RIMS1* gene hypermethylation enables risk stratification of patients with post-*H. pylori* CAG.	Chiang et al.^78^
Multi-omics	In silico (computational)	16S rRNA-seq; TMT-based quantitative proteomics; LC–MS-based untargeted metabolomics	*Coptidis Rhizoma* extract reverses CAG inflammation by modulating NF-κB, PI3K–Akt pathways, and bile acid metabolism, restoring mucosal barrier function.	Ma et al.^3^^,^^79^
	Mouse urine and feces	LC–MS-based metabolomics; 16S rRNA-seq	*Cinnamomi Cortex* alleviates CAG by reshaping gut microbiota, enhancing SCFAs, and suppressing NF-κB/MAPK signaling.	Liu et al.^25^
	Human serum and gastric biopsy	scRNA-seq; WES	H. pylori was associated with UPP1 upregulation via NF-κB activation, suggesting a possible molecular axis linking CAG to intestinal metaplasia.	De Re et al.^80^
	Human gastric mucosa	16S rRNA; ITS sequencing; scRNA-seq; LC–MS-based metabolomics	CagA EPIYA motif variations and VacA s/m genotypes interact with host genetics to determine gastric cancer risk at the chronic atrophic gastritis stage, enabling strain-specific precision prevention.	Zha et al.^81^

Abbreviations: CAG, chronic atrophic gastritis; GWAS, genome-wide association study; SNP array, genome-wide SNP genotyping array; SNP analysis, single nucleotide polymorphism analysis; scRNA-seq, single-cell RNA sequencing; DNBSEQ-G99 RNA sequencing (RNA-seq), RNA sequencing performed on the DNBSEQ-G99 platform developed by MGI; RNA-seq, RNA sequencing; LC-MS/MS, liquid chromatography-tandem mass spectrometry; MALDI-TOF/MS, matrix-assisted laser desorption/ionization time-of-flight mass spectrometry; 2D-DIGE, two-dimensional difference gel electrophoresis; MALDI-TOF/TOF MS, matrix-assisted laser desorption/ionization time-of-flight/time-of-flight mass spectrometry; ˆ1H-NMR, proton nuclear magnetic resonance spectroscopy; GC-TOF-MS, gas chromatography-time-of-flight mass spectrometry; UHPLC-QE-MS, ultra-high-performance liquid chromatography-Q Exactive mass spectrometry; UPLC-Q-TOF/MS, ultra-performance liquid chromatography-quadrupole time-of-flight mass spectrometry; 16S rRNA-seq, 16S ribosomal RNA sequencing; ITS sequencing, Internal Transcribed Spacer sequencing; Targeted LC-MS/MS lipidomics, targeted liquid chromatography-tandem mass spectrometry lipidomics; LC-MS/MS-based glycomics, liquid chromatography-tandem mass spectrometry-based glycomics; MALDI-TOF-MS-based glycomics, matrix-assisted laser desorption/ionization time-of-flight mass spectrometry-based glycomics MALDI-TOF-MS-based glycomics; TMT-based quantitative proteomics, tandem mass tag-based quantitative proteomics; LC-MS-based untargeted metabolomics, liquid chromatography-mass spectrometry-based untargeted metabolomics; LC-MS-based metabolomics, Liquid chromatography–mass spectrometry-based metabolomics; WEG, whole-exome sequencing.

### Genomics

Based on high-density genotyping and genome-wide association studies (GWASs) involving approximately 450,000 participants from the UK Biobank, three novel susceptibility loci for CAG were identified. Transcriptome-wide association studies and eQTL colocalization analyses further implicated C1orf106, PSMA2, and RABGAP1L as potential causal genes, suggesting that these genes may influence susceptibility to CAG through the regulation of gastric epithelial inflammation.^48^ Another study reported that polymorphisms in IL-1B-511 C/T, TNF-α-308 G/A, and IL-10-819 C/T were associated with increased risk of CAG, indicating that imbalance between pro-inflammatory and anti-inflammatory cytokine genes may contribute to disease susceptibility.^49^ In a cohort of 435 patients with autoimmune atrophic gastritis (AAG), the frequency of the HLA-DRB1∗06 allele was significantly higher in females than in males, suggesting potential sex-related differences in genetic susceptibility. However, the study did not assess hormone levels or investigate sex-specific interventions, limiting the interpretation of these findings.^50^ These observations may provide additional insights for risk stratification and individualized management.

Interactions between ERCC6 rs1917799 and ERCC8 rs158572 single nucleotide polymorphisms (SNPs) were associated with reduced risk of CAG, whereas interaction between ERCC6 rs1917799 and ERCC8 rs158916 was linked to lower GC risk. Reduced expression of ERCC6 and ERCC8 proteins in CAG tissues compared with CSG suggests that these genes may participate in CAG development through the modulation of DNA repair pathways.^52^ In addition, tag SNPs of ERCC8 (rs158572 and rs158916) interact with environmental factors such as *H. pylori* infection, smoking, and alcohol consumption, potentially exacerbating gastric mucosal inflammation and injury and increasing the risk of both CAG and GC.^51^ Collectively, these findings support a potential association between ERCC8-related polymorphisms and disease susceptibility, although further validation remains necessary.

### Transcriptomics

Transcriptomics characterizes gene expression profiles and enables the construction of CAG-related expression landscapes, thereby providing insights into underlying molecular mechanisms. By integrating single-cell RNA sequencing (scRNA-seq) with weighted gene co-expression network analysis, 143 shared pathogenic genes associated with CAG were identified, among which GADD45B was highlighted as a potential key regulator. Reduced GADD45B expression was associated with GC occurrence, poor prognosis, and alterations in the immune microenvironment.^53^

In addition, a regulatory network of lncRNA/circRNA-miRNA-mRNA interactions was constructed using DNBSEQ-G99 RNA-seq data from patients with CAG in high-altitude regions. This analysis identified key interaction modules between non-coding RNAs and mRNAs, suggesting their potential relevance in disease regulation.^54^ Whole-transcriptome sequencing further indicated that *H. pylori* infection upregulates HKDC1 expression, which progressively increases during CAG progression. HKDC1 may contribute to gastric mucosal injury and precancerous alterations through the activation of the TGF-β/Smad2/epithelial-mesenchymal transition (EMT) signaling pathway, providing insights into mechanisms associated with the CAG-to-GC transition.^55^ A study published in 2025 utilized scRNA-seq combined with AUCell, SCENIC, and CellChat to construct a 22,511-cell atlas of CAG and early GC, revealing heterogeneity in glutamine metabolism. Epithelial cells in early GC exhibited higher metabolic activity than those in CAG, particularly within the cluster_4 subpopulation. ERO1LB was identified as a potential regulator showing elevated expression in GC and association with poor prognosis. Functional assays suggested that ERO1LB may promote proliferation and migration while inhibiting apoptosis through enhanced glutamine metabolism, indicating its potential relevance in disease progression.^56^

### Proteomics

Recent proteomics studies have shown that, during progression from CAG to GC, ITGB2 expression is markedly upregulated and associated with the activation of NF-κB signaling through the CXCL1-CXCR2 axis. This process may contribute to inflammatory microenvironment remodeling and disease progression toward GPL, suggesting that ITGB2 may represent a candidate biomarker associated with lesion progression.^31^ Additional studies in aged mouse gastric tissues revealed the downregulation of the mitochondrial complex subunit Ndufs8 together with impaired mitophagy. Transplantation of human umbilical mesenchymal stem cells restored Ndufs8 levels, promoted autophagy, and partially reversed gastric gland atrophy, resulting in the improvement of CAG-like pathology in aged mice.^57^ Furthermore, matrix-assisted laser desorption/ionization time-of-flight mass spectrometry (MALDI-TOF/MS) profiling of proteins from *H. pylori* strains in CG-associated patients with GC and CG identified differential proteins, including peroxiredoxin and heat shock protein 70 (HSP70), which may have potential relevance as biomarkers associated with CAG.^58^ Collectively, these findings suggest that differential proteins may contribute to understanding disease progression, although further validation is required.

In AAG, a proteomics study involving 39 patients identified 53 differential proteins in the gastric corpus (covering 67 sampling sites), including 28 upregulated and 39 downregulated proteins. These proteins were associated with pathological processes such as the disruption of the tricarboxylic acid (TCA) cycle, and some overlapped with GC-related proteins, suggesting a potential association between AAG and GC progression.^59^ A study using two-dimensional difference gel electrophoresis (2D-DIGE) combined with MALDI-TOF/TOF MS identified 15 differential proteins, including HSP70 and peroxiredoxin, in AAG-associated *H. pylori* strains, suggesting their potential relevance in AAG pathogenesis.^60^

### Metabolomics

ˆ1H-NMR metabolomics identified five potential biomarkers associated with CAG in plasma (arginine, succinate, and 3-hydroxybutyrate) and urine (α-ketoglutarate and valine), primarily linked to energy metabolism, oxidative stress, and inflammatory pathways.^61^ Dynamic ˆ1H-NMR urinary metabolomics combined with ANOVA-simultaneous component analysis identified 11 candidate biomarkers, including alanine and α-ketoglutarate, which were associated with mitochondrial function, the TCA cycle, and redox pathways.^62^ UPLC-QTOF-MS (ultra-performance liquid chromatography-quadrupole time-of-flight mass spectrometry) analysis further indicated that sphinganine 1-phosphate and several lipid metabolites in tongue coatings are associated with CAG and IM progression, suggesting their potential as non-invasive biomarkers.^63^^,^^64^ Other metabolomics studies have explored pharmacological or experimental interventions in CAG models, implicating dysregulated glucose, lactate, glutamate, lipid, and amino acid metabolism.^65^^,^^66^^,^^67^ These intervention-related findings remain useful for hypothesis generation but require stronger clinical validation before translational conclusions can be drawn.

Collectively, these findings suggest that metabolic alterations are closely associated with CAG progression and may provide potential biomarkers and therapeutic targets, although further validation is required.

### Microbiomics

Microbiome studies have shown that odd-chain fatty acids, including heptadecanoic acid and pentadecanoic acid, are significantly reduced in the feces of patients with CAG and are associated with decreased α-diversity and altered abundances of Blautia and Faecalibacterium, suggesting their potential as non-invasive biomarkers.^68^ 16S rRNA-seq analyses further indicated that the hypochlorhydric environment in AAG may reshape sex-specific microbiota patterns, with males exhibiting reduced microbial diversity and females showing increased abundances of Gemella, Haemophilus, and Neisseria.^69^ In addition, *H. pylori*-associated CAG has been linked to increased relative abundance of Lactobacillus in the gut microbiota of Japanese populations, suggesting microbial dysbiosis associated with disease progression.^70^

Intervention-oriented microbiome studies have reported the modulation of gut microbial composition and related metabolites in CAG models, including changes in choline, trimethylamine, bile acid metabolism, and short-chain fatty acid-producing bacteria.^25^^,^^71^ Placenta-derived PD-MSCs and other mesenchymal stem cells were also associated with reduced inflammation, enhanced autophagy, improved gastric stem cell regeneration, and partial restoration of *H. pylori*-related gut dysbiosis.^72^ Because much of this evidence is preclinical or mechanistic, it should be interpreted as hypothesis-generating rather than clinically established.

Collectively, these findings suggest that microbial dysbiosis is closely associated with CAG progression and may contribute to metabolic and inflammatory alterations within the gastric microenvironment.

### Other omics

Beyond conventional omics, several specialized omics approaches have expanded the scope of CAG research. LC-MS-based lipidomics indicated that Panax notoginseng saponins combined with aspirin modulate arachidonic acid metabolism, potentially through the inhibition of the platelet AA/COX-1/TXB_2_ axis and enhancement of the gastric mucosal AA/PGE_2_ pathway, thereby alleviating aspirin-associated CAG injury.^73^ Glycomics studies using LC-MS/MS and lectin microarrays identified reduced core 1/2 O-glycans and increased sialylated Tn antigens in CAG tissues, alterations that were associated with early tumorigenic changes and EGFR/MAPK pathway activation.^74^ In addition, integrated analyses using lectin microarrays, saliva chips, and lectin blotting demonstrated increased core fucosylation, α2,6-sialylation, and bisecting GlcNAc glycosylation in salivary glycans from patients with CAG. A diagnostic model achieved an AUC of 0.83, suggesting potential utility for non-invasive detection.^75^

Whole-genome methylation microarrays analyzing more than 260,000 CpG sites showed that miR124a-3 methylation reflects the overall gastric mucosal methylation burden, which increases during CAG progression and is associated with elevated GC risk.^76^ Epigenomic studies further indicated that *H. pylori* infection, particularly CagA^+^ strains, is associated with accelerated DNA methylation aging in peripheral blood.^77^ Pyrosequencing analyses of gastric biopsy tissues identified RIMS1 methylation as a potential marker associated with increased annual GC incidence in individuals with open-type atrophy and high methylation levels following *H. pylori* eradication.^78^

### Multi-omics integration

Some preclinical integrative studies combining metabolomics, microbiome profiling, network-based analyses, and animal experiments have linked inflammatory pathways, microbiota-metabolite interactions, bile acid metabolism, and NF-κB/MAPK signaling to CAG-associated mucosal injury.^25^^,^^79^ These studies provide mechanistic clues, but their reliance on model systems and computational target prediction means that they should be considered supportive rather than definitive evidence.

An integrative study combining scRNA-seq and whole-exome sequencing (WES) further revealed a potential mechanism underlying IM progression. *H. pylori* infection was associated with the activation of NF-κB signaling and upregulation of UPP1 expression, which may contribute to epithelial phenotypic transition from CLDN18 to CDH17 during disease progression.^80^ Emerging machine learning approaches may further support multi-omics integration through improved pattern recognition and predictive modeling. A 2025 study integrating microbiomics, metabolomics, and genomics data suggested that variations in CagA EPIYA motifs and VacA s/m genotypes, together with host genetic background, may influence disease progression risk at the CAG stage.^81^

## Spasmolytic polypeptide-expressing metaplasia

SPEM is a metaplastic lesion of the gastric mucosa characterized by aberrant expression of TFF2 in the deep glands of the gastric corpus and is considered a potential precancerous stage in gastric carcinogenesis.^82^ SPEM development is closely associated with gastric mucosal injury, *H. pylori* infection, and reduced acid secretion and is thought to originate from metaplastic transformation of chief or parietal cells during mucosal repair. The frequent detection of SPEM in patients with GC suggests its potential involvement in gastric carcinogenesis. Recent studies have also reported the upregulation of aquaporin 5 (AQP5) in SPEM, which may participate in gastric mucosal injury and repair processes.^83^ The following section summarizes omics-based studies in SPEM and their contributions to understanding this pathological process. SPEM represents an injury-associated metaplastic state characterized by epithelial plasticity and stromal remodeling. Table 4 summarizes omics-based findings in SPEM, highlighting injury-induced cellular plasticity, stromal-epithelial interactions, and microenvironmental remodeling associated with metaplastic transformation.

**Table 4:** Applications and findings of Omics technologies in the research of SPEM

Omics Technology	Sample Information	Specific Technique	Main Findings	Reference
Genomics	Mouse gastric mucosa	WGS	SPEM-related genes were significantly upregulated, but blocking the gastrin receptor failed to suppress SPEM, suggesting that its occurrence may be independent of hypergastrinemia.	Aasarød et al.^84^
	Human blood	SNP analysis	Certain genomic SNPs may increase susceptibility to *H. pylori* infection, thereby promoting the development of SPEM.	Tsai et al.^85^
	Human and mouse gastric mucosa	Gene expression microarray	miR-30a inhibits proliferation and invasion of gastric cancer cells by targeting *ITGA2*, potentially playing a suppressive role in the early stages of SPEM.	Min et al.^86^
	Human gastric mucosa	Targeted exome sequencing	SPEM-specific genetic features revealed genomic associations with gastric cancer (GC) and pyloric gland adenoma.	Srivastava et al.^87^
Transcriptomics	Human gastric mucosa	scRNA-seq	PDGFRα^+^ fibroblasts expand near the epithelium in SPEM regions and may contribute to stromal–epithelial signaling during progression toward dysplasia.	Lee et al.^88^
	Mouse gastric mucosa	scRNA-seq	Injury-activated YAP binds to SPEM gene regulatory elements and upregulates key targets, driving metaplasia and regeneration.	Loe et al.^89^
	Mouse gastric mucosa	scRNA-seq	Post-injury upregulation of *WFDC2* enhances *IL33* expression, driving gastric epithelial conversion to SPEM.	Jeong et al.^10^
	Rat gastric mucosa	scRNA-seq	Zuojin Capsule ameliorates SPEM by regulating abnormal activation of cell cycle–related proteins (e.g., CDK1, CCNB1, CCNA2) and the PI3K-AKT signaling pathway.	Xiong et al.^90^
	Human gastric mucosa	Oligonucleotide microarray	H. pylori infection downregulates AMCase, and may be associated with gastric body atrophy and SPEM through inflammatory and proliferative regulation.	Nookaew et al.^91^
	Human and mouse gastric mucosa	scRNA-seq	Under chronic inflammation, SPEM acquires immune-regulatory gene expression; Tff2^+^Muc6^−^Gif^-^ cells should also be included in the definition of SPEM.	Bockerstett et al.^92^
Proteomics	Human gastric mucosa	2D-DIGE	Twenty-eight differentially expressed proteins identified in SPEM regulate inflammation and cell adhesion, driving its initiation and progression.	Lee et al.^93^
	Mouse gastric mucosa	2D-DIGE	*H. pylori* virulence and inflammation promote the progression of SPEM toward GPL, and 11 SPEM-specific transcripts were identified as biomarkers.	Nomura et al.^94^
	Human gastric mucosa	2D-DIGE	Differential protein expression patterns regulating proliferation, apoptosis, and inflammation in SPEM promote the transition from normal mucosa to dysplasia.	Greengauz-Roberts et al.^95^
Microbiomics	None	16S rRNA-seq; WMS	In AAG-associated SPEM regions, the absence of H. pylori and dysbiosis involving nitrate-reducing and Streptococcus species may be associated with progression toward GC through inflammation and mucosal adaptation.	Isakov^96^
	Mouse gastric mucosa	16S rRNA-seq; WMS	H. pylori and specific microbial consortia remodel the inflammatory–immune microenvironment, potentially contributing to epithelial transformation toward SPEM in INS-GAS mice.	Pinzon-Guzman et al.^97^
Mutil-omics Integration	Human and mouse gastric mucosa	bulk RNA-seq; scRNA-seq; LC-MS/MS	Luteolin binds STAT3 to suppress LCN2 and downstream inflammatory/metaplastic programs, inhibiting SPEM progression. This cross-omics strategy links protein-level target engagement with transcriptional reprogramming, revealing a coherent therapeutic regulatory axis.	Hao et al.^98^
	Human and mouse gastric mucosa	scRNA-seq; Protein-proximity labeling; 3×FLAG purification mass spectrometry	Stratifin may contribute to SPEM by promoting SOX9 upregulation and activating EGFR/ERK signaling, reprogramming chief cells toward proliferative metaplasia. This connects single-cell transcriptional dynamics with pathway activation to define critical transitional states during metaplastic progression.	Won et al.^99^
	Human and mouse gastric mucosa	scRNA-seq; ST; Phosphoproteomics	Interleukins (IL-33, IL-13 and others) orchestrate SPEM through interconnected pathways (such as IL-33/ILC2/IL-13 axis), forming an integrated regulatory circuitry linking immune microenvironment remodeling to epithelial cell fate transitions during metaplastic progression.	Ma et al.^100^

Abbreviations: SPEM, spasmolytic polypeptide-expressing metaplasia; WGS, whole-genome sequencing; SNP analysis, single nucleotide polymorphism analysis; scRNA-seq, single-cell RNA sequencing; 2D-DIGE, two-dimensional difference gel electrophoresis; 16S rRNA-seq, 16S ribosomal RNA sequencing; WMS, whole-metagenome sequencing; ST, Spatial Transcriptomics.

### Genomics

Whole-genome expression profiling, immunohistochemistry, and *in situ* hybridization in H^+^/K^+^-ATPase β-subunit knockout mice revealed marked upregulation of SPEM-associated genes, including Tff2 and Clu. Blockade of the gastrin receptor did not suppress SPEM development, suggesting that SPEM induction may occur independently of hypergastrinemia.^84^ Additional studies indicated that specific SNPs in offspring of patients with GC may increase susceptibility to SPEM following *H. pylori* infection.^85^ MiR-30a has been reported to regulate early intestinal-type GC through the suppression of ITGA2 expression. Reduced miR-30a and elevated ITGA2 levels were observed in SPEM tissues, with a significant negative correlation between the two, suggesting potential involvement of the ITGA2-associated PI3K/Akt pathway in SPEM progression.^86^ Laser capture microdissection (LCM) analysis of SPEM, GC, and pyloric gland adenoma samples further identified SPEM-specific gene expression profiles and suggested molecular links among these lesions.^87^

### Transcriptomics

scRNA-seq analyses revealed expansion of PDGFRα^+^ fibroblasts in SPEM regions adjacent to epithelial cells. These fibroblasts may participate in epithelial transformation through stromal-epithelial interactions and were associated with reduced expression of metaplastic markers such as AQP5 and increased expression of dysplasia-related markers including CEACAM5.^88^ RNA-seq further indicated that activated YAP binds to active chromatin elements of SPEM-related genes following gastric injury, promoting the expression of genes associated with metaplasia, tissue regeneration, and GPL progression.^89^ Additional scRNA-seq studies suggested that SPEM cells may originate from transdifferentiated chief cells and exhibit transcriptional features related to inflammation and tissue repair. WFDC2 has also been associated with epithelial conversion to SPEM, potentially through IL33-mediated M2 macrophage polarization.^10^

Integrated transcriptomic and network pharmacology analyses suggested that Zuojin Capsule may alleviate SPEM-related pathological changes through the regulation of CDK1, CCNB1, CCNA2, and the PI3K-AKT pathway, accompanied by G2/M cell-cycle arrest and reduced tumor-associated macrophage-related proliferation.^90^ Another study reported that *H. pylori* infection is associated with the downregulation of AMCase in atrophic gastric mucosa, suggesting its potential relevance in SPEM and inflammation-associated hyperplasia.^91^ scRNA-seq analyses further indicated that SPEM may follow a conserved transcriptional program regardless of whether it is induced by acute injury or chronic inflammation. Under chronic inflammatory conditions, SPEM acquires additional immune-regulatory gene expression features, suggesting that Tff2^+^Muc6^+^ cells lacking mature chief cell markers such as Gif may represent a broader SPEM-associated cellular state.^92^

### Proteomics

Using LCM combined with 2D-DIGE, researchers analyzed the proteomic composition of SPEM and identified 28 differentially expressed proteins, including annexin A1 and tissue transglutaminase. These proteins are associated with cell adhesion and tissue remodeling, suggesting their involvement in SPEM development.^93^ Subsequent proteomic studies indicated that SPEM formation and its progression toward GPL in *H. pylori*-infected mouse models are associated with bacterial virulence factors and chronic mucosal injury. In addition, microarray screening identified 11 SPEM-specific transcripts, highlighting their potential as diagnostic biomarkers.^94^ Another study using 2D-DIGE combined with cysteine-reactive cyanine dye saturation labeling enhanced the sensitivity of proteomic analysis in LCM-derived samples. This approach revealed differential protein expression patterns related to cell proliferation, apoptosis, and tissue remodeling in SPEM, providing insights into the transition from normal gastric mucosa to dysplasia and suggesting potential diagnostic biomarkers.^95^

### Microbiomics

16S rRNA-seq revealed that, in the gastric mucosa of patients with AAG, SPEM regions are characterized by the depletion of *H. pylori* and enrichment of Neisseria and other nitrate-reducing bacteria. Genera such as Streptococcus may be associated with the the transition of SPEM toward GC through mucosal adaptation and microenvironmental alterations.^96^ Further microbiome analyses in INS-GAS mouse models suggested that *H. pylori* and specific microbial communities may reshape gastric microbiota structure through chronic mucosal perturbation, potentially contributing to epithelial trans differentiation toward SPEM.^97^

Overall, microbiome research in SPEM remains limited. Although certain microbial alterations have been associated with GC, direct mechanistic links to SPEM are not yet well established. Integration of metagenomics with functional studies may help clarify the role of microbiota in SPEM pathogenesis and improve understanding of disease-associated microbial alterations, although further validation is required.

### Multi-omics integration

By integrating transcriptomics (bulk RNA-seq and scRNA-seq data mining) with chemoproteomics (photo-affinity chemoproteomics coupled with LC-MS/MS), together with animal experiments and multiplex immunohistochemistry, one study suggested that luteolin may mitigate gastric precancerous conditions through a target-associated mechanism. Specifically, luteolin was found to interact with STAT3 and was associated with reduced LCN2 expression and attenuation of inflammatory and metaplastic programs, potentially contributing to the inhibition of SPEM progression. This integrative framework links protein-level target engagement with transcriptional alterations, providing insight into potential regulatory mechanisms underlying its observed effects.^101^ Another integrative study combining scRNA-seq with protein-proximity labeling approaches and 3 × FLAG purification mass spectrometry, together with conditional gene knockout mouse models and immunohistochemistry, suggested that Stratifin (SFN) may contribute to SPEM development following acute gastric injury. Mechanistically, SFN was associated with SOX9 upregulation and activation of EGFR/ERK signaling, which may promote lineage transition processes and reprogramming of chief cells toward proliferative metaplasia. These findings connect single-cell transcriptional dynamics with signaling pathway activity and provide insight into transitional cellular states during metaplastic progression.^98^

Furthermore, integrative analyses based on published multi-omics datasets (including scRNA-seq, transcriptomics, and proteomics), together with experimental evidence, suggested that interleukins may participate in SPEM development through inflammatory signaling networks. Key cytokines, including IL-33, IL-13, IL-17A, IL-1β, IL-6, and IL-11, were associated with pathways such as the IL-33/ILC2/IL-13 axis, STAT6, NF-κB, and JAK-STAT signaling. These findings suggest that cytokine-mediated signaling may contribute to interactions between epithelial state transitions and microenvironmental remodeling during SPEM progression.^99^

## Intestinal metaplasia

IM is a pathological alteration characterized by the replacement of the gastric mucosal epithelium with cells that morphologically and functionally resemble those of the small or large intestine. Gastric IM commonly develops in the setting of CAG, whereas esophageal IM is primarily associated with long-term gastroesophageal reflux disease.^100^ IM is widely recognized as a major GPL, and its development is influenced by multiple factors, including *H. pylori* infection, chronic inflammation, and genetic susceptibility.^102^ Although the precise molecular mechanisms underlying IM remain incompletely understood, accumulating evidence supports its important role in gastric carcinogenesis. Further studies are required to clarify its molecular basis, improve risk stratification, and identify potential intervention strategies aimed at reducing progression to GC. IM is characterized by intestinal differentiation and stem-like transcriptional features associated with precancerous progression. Table 5 summarizes omics-based findings in IM, highlighting intestinal differentiation, early driver mutations and clonal expansion, metabolic reprogramming, and microbiome dysbiosis associated with progression toward malignancy.

**Table 5:** Applications and findings of omics technologies in the research of IM

Omics Technology	Sample Information	Specific Technique	Main Findings	Reference
Genomics	Human gastric mucosa	WGS	A small number of somatic mutations appeared at the IM stage, but the genomic structure remained largely stable, suggesting that this precancerous state is driven primarily by epigenetic rather than genomic alterations.	Takeuchi et al.^103^
	Human gastric mucosa	Targeted DNA sequencing; WGS	Driver mutations such as TP53 emerged as early as the IM stage and persisted to accelerate GC progression, indicating that mutations arising during CAG may inform cancer-risk assessment.	Xu et al.^104^
	Human gastric mucosa	WES; WGBS	High-risk IM harbored 26 driver gene mutations (e.g., *ARID1A*, *SOX9*, *FBXW7*), with low mutation burden but evidence of clonal expansion, suggesting partial malignant potential.	Huang et al.^105^
	Human gastric mucosa	WES	“Maintenance-type” driver mutations such as *TP53* appeared during IM and promoted carcinogenesis, whereas IM-enriched mutations like *MUC6* underwent negative selection at the GC stage, indicating dynamic mutation selection during transformation.	Kumagai et al.^106^
Transcriptomics	Human gastric mucosa	scRNA-seq; ST	The intestinal stem-cell compartment of IM harbors 26 driver genes and molecular subtypes associated with inflammation and oral microbiota, associated with risk of progression toward gastric cancer.	Huang et al.^107^^,^^108^; Zhang et al.^109^
	Human gastric mucosa	scRNA-seq; ST	Interactions between PDGFRA^+^ fibroblasts and metaplastic epithelial cells reveal key microenvironmental mechanisms driving IM development and progression.	Tsubosaka et al.^110^
	Human plasma	Plasma transcriptomics	Elevated *TFF3* and *CDH17* levels in IM plasma samples suggest potential as liquid biopsy biomarkers.	Guo et al.^13^
	Human gastric organoids (*in vitro*)	scRNA-seq	The Hippo pathway kinase LATS2 blocks *H. pylori*-induced IM progression by inhibiting YAP/TAZ-mediated epithelial-mesenchymal transition.	Liu et al.^111^^,^^112^
	Human gastric mucosa databases	scRNA-seq	Four ferroptosis-related core genes—*MYB*, *CYB5R1*, *LIFR*, and *DPP4*—were identified as potential diagnostic biomarkers for IM.	Li et al.^14^^,^^15^^,^^42^^,^^43^^,^^44^
	Human gastric mucosa	scRNA-seq; ST	A 26-gene signature (ANPEP, CDH17, OLFM4, DMBT1) characterizing high-risk IM (OLGIM III–IV), shared with intestinal-type gastric cancer. Increased expression of immature stem cell markers during progression suggests their key role in gastric carcinogenesis, offering potential biomarkers for risk stratification.	Huang et al.^19^
	Human gastric mucosa	scRNA-seq; ST	IM is a transitional state driven by activated WNT signaling. UPP1 is progressively upregulated and enriched in H. pylori-positive tissues, where it is associated with intestinal-type differentiation and poor prognosis, suggesting potential relevance to early intervention research.	Chen et al.^20^
Proteomics	Human and mouse gastric mucosa	LC-MS/MS; IP-MS	Loss of *KLHL21* activates the STAT3 pathway via PABPC1-mediated *PIK3CB* translation, promoting IM progression to dysplasia.	Huang et al.^113^
	Human plasma	LC-MS; TMT-based quantitative proteomics	Serum levels of APOA4 and COMP were significantly elevated during IM, suggesting involvement in metaplastic differentiation.	Gong et al.^114^
	Human gastric mucosa	LC-MS	*ITGB2* promotes GC initiation and progression via the CXCL1–CXCR2 axis, providing a novel candidate target for IM.	Shang et al.^31^
	Gastric organoids	Mucinome proteomics	Differentially expressed proteins in IM samples may drive IM-to-GC progression through MAPK signaling and oxidative phosphorylation pathways.	Liu et al.^111^^,^^112^
	Human gastric mucosa	iTRAQ quantitative proteomics; LC-MS/MS	Ribosomal proteins were downregulated at the IM stage, while survival-related proteins (*GSTP1*, *EPCAM*) were upregulated, suggesting potential early GC markers.	Fernández-Coto et al.^115^
	Cell models (*in vitro*)	TMT-based quantitative proteomics; LC-MS/MS	Bile acids disrupt DNA repair and enhance proliferation, contributing to IM and GPL pathogenesis.	Shi et al.^116^
Metabolomics	Human gastric mucosa	UPLC-MS/MS; Q-TOF-MS	During IM-to-GC progression, key metabolites (e.g., N-formylkynurenine, GTP, arginine) changed markedly, and tryptophan and purine metabolism were significantly dysregulated—candidate plasma biomarkers for GC-risk assessment.	Choi et al.^117^
	Human plasma	UPLC-MS/MS	Analysis of 208 metabolites revealed significant alterations in phenylacetylglutamine, tryptophan, and nitrogen metabolism pathways during IM, associated with GC risk.	Huang et al.^118^
	Human tongue coating scrapings	UPLC-MS/MS; Q-TOF-MS	Altered lipid and lipoprotein metabolites in tongue coatings were closely associated with CAG and IM, indicating potential IM progression markers.	You, Lu et al.^63^
	Rat gastric mucosa and body fluids	UPLC-MS; Targeted metabolomics	Huangqi Jianzhong Decoction alleviated IM by modulating gut–thyroid axis–related metabolic pathways and improving thyroid function and metabolic balance.	Xiao et al.^119^
	Rat gastric mucosa	UPLC-MS	DCA-induced IM rats exhibited elevated serum bile acids and increased abundance of *Rikenellaceae RC9*, upregulating lipid digestion-related genes.	Z. Xu et al.^120^
Microbiomics	Human gastric mucosa	16S rRNA-seq	Significant dysbiosis observed in IM and GC, such as the enrichment of *Parvimonas* and depletion of beneficial bacteria.	Sung et al.^121^
	Human gastric mucosa	16S rRNA-seq; WMS	IM mucosa exhibited microbial dysbiosis characterized by increased opportunistic pathogens and reduced probiotics, associated with enrichment in peptidoglycan and purine nucleotide biosynthesis pathways.	Coker et al.^122^
	Mouse gastric mucosa	16S rRNA-seq	DCA-induced IM was accompanied by elevated *Lactobacillus* and *Gemmobacter* abundance and bile acid dysregulation; this imbalance, together with DCA-driven STAT3/KLF5 activation, promoted lesion development.	Jin et al.^123^
	Human oral and gastric samples	Shotgun metagenomic sequencing	Enrichment of oral bacteria (*Peptostreptococcus stomatis*) and gastric bacteria (*Filifactor alocis*) was associated with upregulated LPS synthesis and inflammation pathways, suggesting an oral–gastric microbiome axis in IM initiation.	Wu et al.^124^
	Human and mouse gastric mucosa	Amplicon sequencing	Gastric microbiota from IM or GC patients selectively colonized germ-free mice, inducing precancerous lesions such as dysplasia.	Kwon et al.^41^
	Mouse gastric mucosa	16S rRNA-seq	Long-term vonoprazan exposure caused gastric microbial imbalance, with pathogenic enrichment and decreased *Prevotella*, correlating with increased IM incidence.	Peng et al.^125^
	Human gastric mucosa	16S rRNA-seq; ITS sequencing	Gastric cancer results from synergistic interactions among H. pylori, commensal microbiota, and host immunity, where microbial metabolites and cross-kingdom crosstalk may contribute to IM and malignant transformation.	Mendes-Rocha et al.^126^
Other omics and multi-omics	Mouse gastric mucosa	ChIP-seq	SOX2 loss enhances Wnt signaling and activates CDX2 and other IM-related genes.	Sarkar et al.^127^
	Mouse gastric mucosa	RNA-seq; ChIP-seq	LATS2 loss amplified *H. pylori*-induced EMT signaling, downregulated *CDH1*, upregulated *VIM/MMP*, and accelerated gastric transformation toward IM.	Molina-Castro et al.^128^
	Human gastric mucosa	RNA-seq; WES; DNA methylation microarray	IM organoids were classified into three lineages: early “complete” IM with high *CDX2* expression progressed slowly, while late-stage organoids exhibited *TP53/RUNX3* mutations and intestinal–gastric transcriptional imbalance driving dysplasia.	Yue et al.^129^
	Human gastric mucosa	scRNA-seq; scATAC-seq	Gastric isthmus stem cells can differentiate into SI^+^ metaplastic cells, serving as the origin of IM and subsequent carcinoma.	Liu et al.^130^
	Human gastric mucosa	EPIC array; scRNA-seq; scATAC-seq	A distinct hypermethylation signature in IM, with widespread silencing of tumor suppressor genes and NOS2-mediated epigenetic instability, confirming its precancerous nature.	Takeuchi et al.^103^
	Human gastric mucosa	Visium spatial transcriptomics; RNA-seq; smFISH	LGR5^+^ intestinal stem-like cells localized at IM–tumor junctions; their “intestinal stemness” genes (LGR5, OLFM4, CD44) were associated with high cancer risk and may precede morphological dysplasia.	Huang et al.^107^^,^^108^
	Human and rat gastric mucosa	ChIP-seq; RNA-seq; Genomic methylation sequencing	Key IM-related enhancers (*MYC*, *SOX9*) were activated via demethylation, driving intestinal-type gene expression.	Zhu et al.^131^
	Rat gastric mucosa	UPLC-MS/MS; 16S rRNA-seq	*Jianwei Xiaoyan Granules* reversed CAG-associated IM by inhibiting the HIF-1α–VEGF pathway and restoring gastric epithelial differentiation.	Liu et al.^111^^,^^112^

Abbreviations: IM, Intestinal Metaplasia; WGS, whole-genome sequencing; WGBS, whole-genome bisulfite sequencing; WES, whole-exome sequencing; scRNA-seq, single-cell RNA sequencing; ST, spatial transcriptomics; LC-MS/MS, liquid chromatography-tandem mass spectrometry; IP-MS, immunoprecipitation–mass spectrometry; LC-MS, liquid chromatography-mass spectrometry; 16S rRNA-seq, 16S ribosomal RNA sequencing; TMT-based quantitative proteomics, tandem mass tag-based quantitative proteomics; Q-TOF-MS, quadrupole time-of-flight mass spectrometry; UPLC-MS/MS, ultra-performance liquid chromatography-tandem mass spectrometry; UPLC-MS, ultra-performance liquid chromatography-mass spectrometry; WMS, whole-metagenome sequencing; ITS sequencing, Internal transcribed spacer sequencing; ChIP-seq, chromatin immunoprecipitation sequencing; RNA-seq, RNA sequencing; scATAC-seq, single-cell assay for transposase-accessible chromatin using sequencing; EPIC array, Infinium MethylationEPIC BeadChip array; smFISH, single-molecule FISH.

### Genomics

IM exhibits premalignant characteristics and the potential for progression toward GC. Genomic studies have demonstrated that IM is associated with both genetic and epigenetic alterations. Whole-genome sequencing (WGS) studies identified a relatively limited number of somatic mutations at the IM stage, while overall genomic stability remained largely preserved. These findings suggest that epigenetic alterations may play a more prominent role than genetic mutations during early IM progression.^103^ Multi-regional WES of 330 paired IM-tumor samples from 93 patients with GC revealed that “maintenance-type” driver mutations, such as TP53, may emerge at the IM stage and are associated with malignant progression. In contrast, IM-enriched mutations, including MUC6, appear to undergo negative selection during cancer development, suggesting dynamic mutational selection during gastric carcinogenesis.^104^ Integrated analyses combining WES and whole-genome bisulfite sequencing identified 26 driver gene mutations (e.g., ARID1A, SOX9, and FBXW7) in high-risk IM. Despite the relatively low mutational burden, evidence of clonal expansion suggests that a subset of IM cells may already possess oncogenic potential.^105^ WES analyses further indicated that progression from IM to GC is associated with mutations in CDH1, TP53, and RHOA, together with the amplification of chromosome 20q, providing potential molecular insights for risk stratification and early intervention.^106^

### Transcriptomics

Transcriptomic approaches have been widely applied to identify differentially expressed genes in IM and evaluate the risk of progression to GC. scRNA-seq and spatial transcriptomics (ST) studies revealed a distinct cellular compartment within IM tissues enriched in intestinal stem cell-like populations. These analyses identified 26 driver genes and molecular subtypes associated with inflammation and oral microbiota, providing insight into malignant progression risk.^107^^,^^109^ Fibroblasts expressing PDGFRA, BMP4, and WNT5A were shown to interact with metaplastic epithelial cells, suggesting that stromal-epithelial crosstalk may contribute to IM development and progression.^110^ Plasma transcriptomic profiling indicated that the goblet cell marker TFF3 and the intestinal epithelial marker CDH17 are elevated in patients with IM, supporting their potential as liquid biopsy biomarkers for IM and early GC.^13^ Mechanistic studies further suggested that the Hippo pathway kinase LATS2 inhibits YAP/TAZ-mediated EMT, thereby suppressing *H. pylori*-associated IM progression.^111^ In addition, integration of public transcriptomic datasets with machine learning approaches identified four ferroptosis-related hub genes—MYB, CYB5R1, LIFR, and DPP4—as candidate diagnostic biomarkers for IM.^43^

In 2025, integrative analyses combining ST and scRNA-seq in an OLGIM-staged IM cohort identified a 26-gene signature associated with high-risk IM (OLGIM III-IV). This signature, localized to metaplastic lesions, included both mature intestinal markers (ANPEP and CDH17) and immature stem cell markers (OLFM4 and DMBT1) and overlapped with intestinal-type GC. Single-cell and spatial analyses further demonstrated increased expression of immature intestinal stem cell markers during disease progression, suggesting their potential relevance to gastric carcinogenesis and risk stratification.^19^ A 2026 study integrating single-cell and ST across 18 gastric mucosal samples characterized IM as a transitional state associated with WNT signaling activation. UPP1 expression progressively increased and was enriched in *H. pylori*-positive samples, while UPP1-high cells exhibited enhanced intestinal differentiation features. Functional and clinical analyses suggested that UPP1 may contribute to intestinal-type differentiation and is associated with poor prognosis in GC.^20^

Emerging machine learning approaches may further facilitate multi-omics integration and predictive modeling across different stages of disease progression.

### Proteomics

Mass spectrometry-based proteomic analyses showed that loss of KLHL21 is associated with the activation of the STAT3 signaling pathway via the PABPC1-PIK3CB axis, which may contribute to progression from IM to dysplasia.^113^ Serum proteomic profiling using LC-MS/MS demonstrated significant upregulation of intestinal epithelial-associated proteins APOA4 and COMP in patients with IM, supporting their potential as candidate biomarkers.^114^ TMT-labeled proteomic analysis further revealed increased ITGB2 expression during the IM stage, which was associated with GPL progression through the CXCL1-CXCR2 axis.31 In addition, mucoproteomic sequencing identified elevated levels of MUC17, CDA, TRIM15, and TBX3, suggesting their possible involvement in IM initiation and progression through MAPK signaling and oxidative phosphorylation.^111^

Quantitative proteomics using iTRAQ labeling revealed downregulation of ribosomal proteins together with the upregulation of cell survival-related proteins, including GSTP1 and EPCAM, during the IM stage, suggesting their potential as early indicators of GC progression.^115^ Quantitative LC-MS/MS analysis also showed that DCA (deoxycholic acid) induces apoptosis in GES-1 cells and alters the expression of proteins associated with DNA repair, cell-cycle regulation, proliferation, and adhesion. These findings suggest that bile acids may contribute to IM and GPL pathogenesis through the modulation of DNA repair capacity and cellular proliferation.^116^

Collectively, these studies provide proteomic insights into molecular alterations associated with gastric carcinogenesis, although further experimental validation is still required.

### Metabolomics

Metabolomic studies have revealed distinct metabolic differences between IM and GC, with key metabolites such as N-formylkynurenine, GTP, and arginine showing significant alterations. Dysregulation of tryptophan and purine metabolism suggests their potential relevance to progression from IM to GC.^117^ Complementary plasma metabolomics using targeted LC-MS analysis identified significant alterations in metabolites related to phenylacetylglutamine, tryptophan metabolism, and nitrogen metabolism during the IM stage, supporting their possible utility in early detection.^118^ In addition, tongue-coating lipidomics demonstrated that metabolites including 5,6-dihydroxyindole, α-carboxy-δ-decalactone, sphinganine-1-phosphate, and trimethylaminopropanol were positively associated with IM, whereas 1-methyladenosine showed a negative association, suggesting the potential utility of non-invasive lipidomic markers for IM monitoring.^63^

Untargeted serum metabolomics further indicated that Huangqi Jianzhong Tang may modulate intestinal-thyroid axis-related metabolic pathways, normalize altered metabolites in IM model rats, and alleviate IM-associated pathological changes.^119^ Targeted metabolomics of serum bile acids showed that DCA-induced IM in rats was associated with increased levels of DCA, taurocholic acid, and taurodeoxycholic acid, which correlated with the abundance of Rikenellaceae RC9 and expression of lipid metabolism-related genes (Fabp1 and Dgat1), suggesting a potential DCA-microbe-gene regulatory axis.^120^

### Microbiomics

Microbiome studies have improved understanding of IM and early GC risk. 16S rRNA-seq revealed that one year after *H. pylori* eradication, oral-derived bacteria such as Peptostreptococcus, Streptococcus, and Parvimonas were enriched, whereas beneficial bacteria including Faecalibacterium were reduced, which was associated with the occurrence and persistence of IM.^121^ Integration of 16S rRNA data from multiple cohorts demonstrated gastric microbiota dysbiosis during the IM stage, characterized by the enrichment of opportunistic pathogens (e.g., Veillonella and Parvimonas) and depletion of potential probiotics (e.g., Bifidobacterium), together with increased peptidoglycan and purine biosynthesis pathways.^122^ Additional studies showed that DCA-induced IM is associated with increased gastric abundances of Lactobacillus and Gemmobacter accompanied by dysregulated bile acid metabolism. Combined with DCA-mediated activation of the STAT3/KLF5 pathway, these alterations may contribute to lesion progression.^123^

Specific oral-gastric microbial communities, including Peptostreptococcus stomatis, Johnsonella ignava, and Filifactor alocis, were enriched in IM tissues and associated with inflammatory pathway activation, suggesting a potential role in IM development.^124^ Animal studies further demonstrated that IM-associated microbiota can colonize germ-free mouse stomachs and induce GPL, supporting their possible functional relevance in disease progression.^41^ Long-term administration of vonoprazan in mice was also associated with gastric microbial dysbiosis characterized by the enrichment of potential pathogens and depletion of beneficial bacteria, together with increased IM incidence.^125^ Integrative analyses combining 16S rRNA, ITS sequencing (internal transcribed spacer sequencing), and metagenomics further suggested that IM represents a transition stage shaped by interactions among *H. pylori*, bacterial, fungal, and viral communities, as well as host immunity. Microbiota-associated metabolites and cross-kingdom interactions may contribute to epithelial reprogramming and microenvironmental remodeling during IM, although additional mechanistic studies remain necessary.^126^

### Other omics and mutil-omics integration

Epigenetic and multi-omics studies have provided further insight into mechanisms associated with IM initiation and progression. ChIP-seq (chromatin immunoprecipitation sequencing) analyses showed that loss of SOX2 is associated with enhanced Wnt signaling and activation of CDX2 and other IM-related genes, suggesting that SOX2 may function as an epigenetic regulator suppressing GPL development.^127^ Integrative analyses combining DNA methylation arrays and scRNA-seq further indicated that LATS2 deficiency may amplify *H. pylori*–associated EMT signaling, accompanied by CDH1 downregulation and VIM/MMP upregulation, thereby contributing to IM-associated epithelial reprogramming.^128^

Another study integrating scRNA-seq, DNA methylation arrays, and WES revealed cellular heterogeneity, lineage evolution, and genetic features of human gastric IM organoids. Early “complete-type” IM characterized by high CDX2 expression appeared to progress more slowly, whereas advanced IM exhibited TP53 and RUNX3 mutations together with imbalances in intestinal-gastric transcriptional programs, suggesting increased risk of dysplasia and malignant progression.^129^ Single-cell transcriptomics combined with single-cell assay for transposase-accessible chromatin sequencing including single-cell assay for transposase-accessible chromatin using sequencing, like single-cell assay for transposase-accessible chromatin using sequencing (scATAC-seq) further suggested that gastric isthmus stem cells may differentiate into SI^+^ metaplastic cells, potentially representing a cellular origin of IM and subsequent carcinoma.^130^ Integrated analyses of DNA methylation, ChIP-seq, and related technologies also identified a hypermethylation signature in IM characterized by widespread silencing of tumor suppressor genes and NOS2-associated epigenetic instability, supporting the precancerous nature of IM.^103^

Spatial and integrative multi-omics analyses have further refined current understanding of IM pathogenesis. Integration of ST, scRNA-seq, and single-molecule fluorescence *in situ* hybridization (smFISH) identified 26 highly expressed genes in IM lesions associated with high-risk precursor cell populations in intestinal-type GC. LGR5^+^ intestinal stem-like regions at the IM-tumor interface exhibited an “intestinal stem” gene signature (LGR5/OLFM4/CD44), suggesting possible involvement in malignant progression.^108^ Combined ChIP-seq, RNA-seq, and whole-genome methylation sequencing enabled construction of enhancer-promoter interaction maps, revealing that IM-associated enhancers such as MYC and SOX9 may be activated through demethylation and contribute to intestinal phenotype gene expression.^131^ Integrative analyses combining *H. pylori*-related LC-Q-TOF-MS/MS metabolomics, 16S rRNA-seq, and RNA-seq further suggested that Jianwei Xiaoyan Granules may alleviate CAG-associated IM through the modulation of the HIF-1α-VEGF signaling pathway, providing mechanistic insight into potential traditional Chinese medicine-related interventions.^112^

However, most current integrative analyses remain largely descriptive, with limited mechanistic resolution regarding how different omics layers converge on key regulatory pathways. In addition, heterogeneity across datasets and lack of large-scale validation may affect the robustness and generalizability of these findings. Recent integrative multi-omics studies have further characterized the dynamic evolutionary trajectory of IM. Accumulating evidence suggests that epigenetic reprogramming, expansion of stem-like cell populations, and microenvironmental remodeling are associated with progression toward dysplasia. Spatial and single-cell analyses further indicate that intestinal stem-like cells with high-risk transcriptional signatures may emerge prior to overt morphological changes, supporting their potential relevance in early gastric carcinogenesis.

## Dysplasia

Dysplasia represents a pivotal precancerous stage in GC development, characterized by morphological and functional abnormalities of gastric mucosal epithelial cells, including cellular pleomorphism, abnormal mitoses, and disrupted tissue architecture. Its development is influenced by multiple factors, among which *H. pylori* infection plays a central role. Virulence factors such as CagA and VacA are associated with chronic inflammation and mucosal injury, thereby contributing to lesion progression. At the molecular level, genetic alterations including CDX2 and TPT1 are associated with dysplasia formation and IM, while environmental and lifestyle factors such as smoking and vitamin D deficiency may further increase susceptibility.^132^ The following section summarizes recent multi-omics advances related to dysplasia and their relevance to disease progression and risk assessment. Dysplasia represents a stage of early neoplastic transformation characterized by progressive molecular and cellular abnormalities. Table 6 summarizes omics-based findings in dysplasia, including the accumulation of driver mutations, epigenetic silencing of tumor suppressor genes, activation of oncogenic signaling pathways, metabolic reprogramming, and progressive microbial dysbiosis.

**Table 6:** Applications and findings of omics technologies in the research of Dysplasia

Omics technology	Sample information	Specific technique	Main findings	Reference
Genomics	Human gastric mucosa	WES; WGBS; EBV genome sequencing;	In EBV-positive GPL, viral genome integration and host CpG island methylation accumulated significantly at the dysplasia stage, leading to inactivation of tumor suppressor genes such as *TP53* and *CDKN2A*.	Chen et al.^133^
	Mouse gastric mucosa	NanoString nCounter gene expression analysis	Persistent *H. pylori* infection accelerates gastric dysplasia in mice and induces key mutations such as *Trp53* and *Apc* inactivation and *Ctnnb1* activation, revealing the genomic basis of infection-driven malignant transformation.	O'Brien et al.^134^
	Human gastric mucosa	Targeted deep DNA sequencing	*TP53* mutation and *CDH1* heterozygous deletion are early key events in intestinal-type intramucosal neoplasia, driving dysplasia development.	Rokutan et al.^135^
Transcriptomics	Human gastric mucosa	scRNA-seq	Interaction between fibroblasts and gastric metaplastic cells activates pathways such as Wnt5a, promoting the transition from IM to dysplasia and highlighting the pivotal role of stroma–epithelial interaction in GPL.	Lee et al.^88^
	Human gastric mucosa	Microarray analysis	During the progression from normal mucosa to dysplasia and gastric cancer, Wnt/β-catenin, NF-κB, and EMT pathways are gradually activated, with key genes such as *MMP7*, *SOX9*, and *CLDN1* identified.	Li et al.^136^
	Mouse gastric mucosa	scRNA-seq	PARD and IFN-γ synergistically activate pro-inflammatory and stemness pathways, driving progenitor cell dysplasia and promoting GC development in mice.	Zuo et al.^137^
	Mouse gastric mucosa	cRNA-seq; ST	A pro-tumorigenic fibroblast subpopulation highly expressing *FAP*, *POSTN*, and *WNT5A* emerges during dysplasia, promoting tumor progression via Wnt and EMT activation.	Contreras-Panta et al.^138^
	Human gastric mucosa	scRNA-seq	*TFF1* loss activates the NF-κB pathway, induces inflammatory gene expression, and promotes dysplasia and tumorigenesis, revealing the role of inflammation in GPL.	Soutto et al.^139^
Proteomics	Human and mouse gastric mucosa	Mass spectrometry-based proteomics	*KLHL21* deficiency disrupts STAT3 balance, promotes gastric dysplasia, and normally maintains signaling homeostasis by degrading STAT3 inhibitors—suggesting its potential tumor-suppressive role.	Huang et al.^113^
	Human gastric mucosa	iTRAQ labeling proteomics	Oncogenic *H. pylori* infection disturbs gastric proteome, upregulating IL-1β, NF-κB, and proliferation markers associated with dysplasia, indicating inflammation- and proliferation-driven GPL progression.	Noto et al.^140^
	Mouse gastric mucosa	LC-MS/MS	During the transformation from dysplasia to early gastric cancer, proteins such as ANXA2 and S100A11 are significantly elevated, serving as potential markers of malignant transformation.	Li et al.^17^
	Human plasma	DIA proteomics; LC-MS/MS	Plasma proteome shows dynamic changes from normal mucosa to cardia cancer, identifying dysplasia-specific differential proteins as potential biomarkers for early detection of high-risk lesions.	Gu et al.^141^
	Human serum	Olink® proteomic platform	Serum levels of HSP60, 14-3-3ζ, and Annexin A2 increase with cardia dysplasia, showing potential for early diagnosis.	Li et al.^14^^,^^15^^,^^42^^,^^43^^,^^44^
Metabolomics	Human gastric mucosa	NMR	Dysplastic tissue exhibits enhanced glycolysis and abnormal glutamine and choline metabolism, revealing oncogenic metabolic features.	Gu et al.^142^
	Human gastric mucosa	GC-TOF-MS	Dysplasia and GC stages show disrupted glycolysis, amino acid, and fatty acid metabolism, which may serve as early diagnostic metabolic biomarkers.	Yu et al.^37^
	Human gastric mucosa	UPLC-QTOF-MS	Key dysplasia-associated metabolites (e.g., bile acids, glutamate) and pathway alterations were identified along the Correa cascade, suggesting that metabolic reprogramming may contribute to GC initiation.	Kuligowski et al.^143^
Microbiomics	Human feces	16S rRNA-seq	*Streptococcus anginosus* and *S. constellatus* were significantly enriched in feces of dysplasia patients, representing potential non-invasive microbial biomarkers for early screening.	Zhou et al.^144^
	Human gastric mucosa and feces	16S rRNA-seq	*H. pylori* infection increases the diversity of non-*H. pylori* genera (e.g., *Prevotella*, *Streptococcus*) and dysplasia risk, linking microbial dysbiosis to GPL.	Guo et al.^145^
	Human gastric mucosa	16S rRNA-seq	Gastric mucosal microbiota diversity decreases in dysplasia; *Nitrospirae* and *Aggregatibacter actinomycetemcomitans* are markedly enriched.	Zhou et al.^146^
	Human gastric mucosa	16S rRNA-seq	After H. pylori eradication, gastric microbial diversity decreases, pro-inflammatory taxa such as Nitrosospira are enriched, and dysplasia risk may increase, suggesting microecological imbalance may contribute to carcinogenesis.	Piscione et al.^147^
	Human gastric mucosa	16S rRNA-seq	*cagA*^*+*^ strains activate the IL-1β/NLRP3 pathway, inducing innate immune dysregulation and driving gastric dysplasia.	Gobert et al.^148^
	Human gastric mucosa	16S rRNA-seq; ITS2 rRNA sequencing	GC microenvironment exhibits fungal dysbiosis with altered diversity and enriched pro-inflammatory taxa during the CAG-to-GC transition, driving gastric carcinogenesis via fungal-bacterial cross-kingdom interactions.	Zhang et al.^149^
Multi-omics	Human gastric mucosa	WGS; scRNA-seq; Plasma cfDNA targeted sequencing	Identified ten key genes closely related to gastric dysplasia, showing potential for early detection and progression monitoring.	Long et al.^150^
	Human and mouse gastric mucosa	16S rRNA-seq; scRNA-seq	*CagA* activates the NF-κB/STAT3 axis, driving an inflammation–tumor positive feedback loop that promotes gastric dysplasia.	Suzuki et al.^151^

Abbreviations: WES, whole-exome sequencing; WGBS, Whole-genome bisulfite sequencing; scRNA-seq, single-cell RNA sequencing; ST, spatial transcriptomics; LC-MS/MS, Liquid chromatography-tandem mass spectrometry; DIA proteomics, Data-Independent Acquisition proteomics; NMR, Nuclear magnetic resonance spectroscopy; GC-TOF-MS, gas chromatography-time-of-flight mass spectrometry; UPLC-QTOF-MS, ultra-performance liquid chromatography-quadrupole time-of-flight mass spectrometry; 16S rRNA-seq, 16S ribosomal RNA sequencing; ITS2 rDNA sequencing, Internal transcribed spacer 2 ribosomal DNA sequencing; WGS, whole-genome sequencing.

### Genomic

Genomic studies have provided important insights into mechanisms associated with dysplasia progression. In EBV-positive GPL, viral genome integration and host CpG island methylation accumulate during the dysplastic stage and are associated with the inactivation of tumor suppressor genes such as TP53 and CDKN2A, suggesting potential early events in malignant transformation.^133^ WGS studies further indicated that persistent *H. pylori* infection is associated with accelerated dysplasia development in mouse models, accompanied by inactivating mutations in Trp53 and Apc, as well as activating mutations in Ctnnb1.^134^ Moreover, TP53 mutations and heterozygous loss of CDH1 have been identified as early genetic events in intestinal-type intramucosal gastric tumors, supporting their involvement in dysplasia development.^135^

### Transcriptomics

scRNA-seq analyses suggest that interactions between fibroblasts and gastric metaplastic epithelial cells are associated with the activation of signaling pathways such as Wnt5a, potentially facilitating the transition from metaplasia to dysplasia and supporting the role of stromal-epithelial crosstalk in GPL progression.^88^ Transcriptomic studies further indicate that progression from normal gastric mucosa to moderate-to-severe dysplasia and eventually GC is accompanied by the progressive activation of pathways including Wnt/β-catenin, NF-κB, and EMT, with genes such as MMP7, SOX9, and CLDN1 identified as candidate early biomarkers.^136^

In addition, PPARD has been reported to cooperate with IFN-γ in regulating pro-inflammatory and stem cell-related pathways, which may contribute to dysplasia development in gastric progenitor cells.^137^ Single-cell analyses also identified a fibroblast subpopulation characterized by high expression of FAP, POSTN, and WNT5A that is associated with lesion progression, potentially through the activation of Wnt- and EMT-related signaling pathways.^138^ Another study suggested that loss of TFF1 is associated with activation of NF-κB signaling and induction of inflammatory gene expression, which may contribute to dysplasia and tumorigenesis.^139^

### Proteomics

Proteomic analyses suggest that loss of KLHL21 is associated with the dysregulation of STAT3 signaling, which may contribute to gastric dysplasia, whereas KLHL21 may help maintain signaling homeostasis, supporting a potential tumor-suppressive role.^115^ In addition, *H. pylori* infection has been associated with alterations in the gastric proteome, including increased expression of inflammatory mediators such as IL-1β, proteins involved in NF-κB signaling, and proliferation-related markers, suggesting that inflammatory dysregulation and abnormal cellular proliferation may contribute to GPL progression.^140^ During the transition from gastric dysplasia to early GC, proteins such as ANXA2 and S100A11 were found to be upregulated, indicating their potential relevance as biomarkers for malignant transformation.^17^

Plasma proteomics studies further demonstrated dynamic alterations across disease stages, including normal mucosa, atrophy, dysplasia, and GC, with specific protein changes observed during the dysplasia stage, suggesting possible candidates for early detection of high-risk lesions.^141^ In addition, serum levels of HSP60, 14-3-3ζ, and Annexin A2 increased progressively with dysplasia severity, supporting their potential utility as early diagnostic biomarkers.^44^

### Metabolomics

Metabolomic studies have shown that dysplastic tissues exhibit enhanced glycolysis, disrupted glutamine metabolism, and altered choline metabolism, reflecting pro-tumorigenic metabolic characteristics.^142^ GC-TOF-MS analyses further demonstrated significant dysregulation of glycolysis, amino acid, and fatty acid metabolism during dysplasia and GC stages, suggesting that these metabolic alterations may have value in early disease detection.^37^ In addition, UPLC-MS (ultra-performance liquid chromatography-mass spectrometry)-based metabolomics identified key dysplasia-associated metabolites, including bile acids and glutamate, together with altered metabolic pathways across the Correa cascade, supporting the involvement of metabolic reprogramming in gastric carcinogenesis.^144^

### Microbiomics

Microbial dysbiosis is closely associated with the dysplastic state and may contribute to lesion progression. 16S rRNA-based studies identified significant enrichment of Streptococcus anginosus and Streptococcus constellatus in fecal samples, which were associated with gastric mucosal dysplasia and may serve as non-invasive biomarkers for early GC detection.^145^ *H. pylori* infection has also been associated with increased diversity of non-*H. pylori* gastric microbiota, including Prevotella and Streptococcus species, suggesting a relationship between microbial imbalance and dysplasia risk.^143^ Patients with dysplasia additionally exhibit reduced gastric mucosal microbial diversity accompanied by increased abundance of Nitrospirae and Aggregatibacter actinomycetemcomitans.^146^ Following *H. pylori* eradication, microbial diversity may decline while pro-inflammatory taxa become enriched, potentially contributing to dysplasia risk.^147^ Moreover, certain *H. pylori* strains (e.g., CagA^+^) can activate aberrant innate immune signaling via the IL-1β/NLRP3 pathway, further supporting the role of microbiota-immune interactions in GPL pathogenesis.^148^ Studies integrating ITS2 rDNA sequencing (internal transcribed spacer 2 ribosomal DNA sequencing) and 16S rRNA-seq further revealed progressive fungal dysbiosis across the gastritis-carcinoma sequence, which becomes more pronounced at the dysplasia stage. These findings suggest that fungi may participate in lesion progression through cross-kingdom interactions with bacteria and modulation of the gastric microenvironment.^150^

### Multi-omics integration

Integration of WGS and RNA-seq enabled identification of ten key genes dynamically associated with gastric dysplasia, suggesting their potential relevance as biomarkers for early detection and disease monitoring. This integrative strategy linked genomic alterations with transcriptional reprogramming, indicating that dysplasia-associated mutations may contribute to coordinated gene expression changes during disease progression.^151^ Another study combining scRNA-seq with 16S rRNA-seq revealed a microbiota-host interaction network in gastric epithelial dysplasia. Specifically, *H. pylori* CagA was associated with the activation of the NF-κB/STAT3 signaling axis and induction of pro-inflammatory transcriptional programs at the single-cell level, while also establishing interactions between microbial stimulation and host inflammatory responses.^152^ However, these integrative analyses remain largely descriptive, with limited mechanistic insight into how different omics layers converge on key regulatory pathways. In addition, inconsistencies across datasets and the lack of large-scale validation may affect the robustness of these findings.

## Overall overview of stage-specific omics landscapes across the gastritis-to-carcinoma sequence

To provide a systematic overview of omics-based research in CG and GPL, Table 1 summarizes the major omics technologies and their application characteristics, including genomics, transcriptomics, proteomics, metabolomics, and microbiome analysis, highlighting their respective advantages and limitations in studying gastric disease progression. Tables 2, 3, 4, 5, and 6 further summarize representative findings across different stages of the gastritis-carcinoma sequence, including CNAG, CAG, SPEM, IM, and dysplasia.

Collectively, these studies reveal stage-specific molecular features and progressively evolving regulatory patterns. In CNAG and early gastritis, omics analyses primarily indicate inflammatory activation and microbial perturbations, with transcriptomic and microbiome studies showing early immune responses and *H. pylori*-associated dysbiosis. As the disease progresses to CAG, multi-omics data demonstrate enhanced immune dysregulation, epithelial atrophy, and metabolic alterations, including changes in lipid metabolism and mitochondrial function. In SPEM, integrative omics analyses emphasize injury-associated epithelial plasticity and microenvironmental remodeling. Single-cell transcriptomic studies reveal expansion of epithelial and stromal populations, including PDGFRα^+^ fibroblasts, together with the activation of YAP signaling and inflammatory pathways associated with metaplastic transformation. Proteomic studies additionally support the involvement of inflammatory and cell adhesion-related processes, whereas microbiome analyses suggest that microbial dysbiosis may participate in immune remodeling and disease progression. During progression to IM, transcriptomic and epigenetic analyses indicate the activation of intestinal differentiation programs, including CDX2-associated pathways, accompanied by metabolic reprogramming and microbiome alterations. Proteomic and metabolomic studies further reveal disturbances in energy metabolism and bile acid-related pathways, reflecting progression toward a pre-neoplastic state.

As lesions progress to dysplasia, multi-omics studies indicate increasing neoplastic features, including the accumulation of driver alterations such as TP53 mutation and CDH1 loss, epigenetic silencing of tumor suppressor genes, and activation of oncogenic pathways including Wnt/β-catenin, NF-κB, and EMT signaling. Transcriptomic and single-cell analyses further demonstrate emergence of pro-tumorigenic fibroblast subpopulations and enhanced inflammatory-stemness signaling, whereas proteomic and metabolomic studies suggest increased proliferative activity, glycolytic reprogramming, and amino acid metabolic dysregulation. In parallel, microbiome analyses reveal reduced microbial diversity and enrichment of pro-inflammatory taxa, supporting a potential association between microbial dysbiosis and malignant progression. Across the gastritis-to-carcinoma sequence, microbial alterations evolve from *H. pylori*-associated dysbiosis in early gastritis toward more diverse oral-associated microbial communities during IM and dysplasia, suggesting a potential role for microbiota in disease progression.

Together, these findings support a progressive transition from inflammation to metaplasia and neoplastic transformation, involving coordinated alterations in epithelial biology, immune responses, metabolism, and microbial communities. These integrated observations provide a systems-level perspective on gastric carcinogenesis and may help inform future mechanistic and translational studies.

### Breakthrough contributions of omics technologies in CG and GPL research

Over the past decade, omics technologies have substantially advanced CG and GPL research by providing panoramic, dynamic, and increasingly high-resolution insights into disease progression. Multi-omics integration enables the combined analysis of genomic, transcriptomic, proteomic, metabolomic, microbial, and epigenetic layers, facilitating a more comprehensive understanding of the gastritis-to-carcinoma sequence. Single-cell and spatial omics approaches further improve resolution by enabling characterization of cellular heterogeneity, lineage relationships, and microenvironmental alterations. Dynamic analyses partially overcome the limitations of static models by allowing temporal assessment of disease-associated changes. In addition, emerging computational approaches, including machine learning-based cross-omics analyses, have shown potential for identifying candidate molecular signatures from complex datasets. However, these signatures should be interpreted cautiously because clinical applicability remains limited by cohort size, data heterogeneity, and insufficient external validation.

CG and GPL, as critical stages in GC progression, are increasingly being investigated at the mechanistic level. Genomic studies have identified susceptibility loci, including HLA polymorphisms associated with CAG risk,^153^ as well as methylation signatures that may serve as early biomarkers for GC.^76^ Interactions between genetic variants (e.g., ERCC8 rs158572) and environmental factors, including *H. pylori* infection and smoking, may further refine risk stratification for high-risk populations.^52^ Transcriptomic analyses reveal dynamic alterations in coding and non-coding RNA expression and highlight their potential regulatory roles in disease progression.^54^

Proteomics and metabolomics provide insights into protein and metabolite alterations associated with disease evolution. Proteomic profiling has identified differentially expressed proteins in CAG that may represent candidate therapeutic targets.^59^ Dysregulation of the KLHL21-STAT3 axis has also been associated with dysplasia progression,^20^ while altered gastric mucin O-glycosylation identified through glycoproteomics suggests additional candidate intervention targets.^73^ Metabolomic studies further indicate metabolic reprogramming during gastric carcinogenesis, including alterations in energy metabolism, oxidative stress, and inflammatory pathways.^61^ Certain lipid metabolites in tongue coatings, such as sphingosine-1-phosphate, may serve as potential non-invasive biomarkers, although additional validation is required.64 Microbiome studies similarly demonstrate substantial microbial dysbiosis across CG and GPL stages.^26^ *H. pylori* infection, together with broader microbial alterations, may contribute to lesion progression. For example, recolonization by oral-associated bacteria such as Peptostreptococcus following *H. pylori* eradication has been associated with IM progression.^124^ In addition, microbiota-bile acid metabolic interactions, including the DCA—Rikenellaceae axis,^120^ provide insights into potential regulatory mechanisms and therapeutic strategies.^70^

Integrated multi-omics analyses suggest that progression from CG to GPL involves coordinated disturbances across host genetic, microbial, and metabolic networks. *H. pylori* infection may contribute to lesion progression through combined effects on DNA methylation and inflammatory signaling. Although intervention-oriented studies have reported the modulation of related pathways, current evidence remains preliminary and requires validation in larger, well-controlled human studies.

Collectively, multi-omics studies across the gastritis-carcinoma sequence reveal several recurring patterns. First, disease progression involves coordinated alterations across multiple molecular layers, in which genomic and epigenetic perturbations are associated with downstream transcriptional, proteomic, and metabolic changes. Second, interactions among epithelial cells, immune responses, and microbial dysbiosis represent common features throughout disease progression. Third, integrative analyses identify key regulatory pathways related to inflammation, metabolism, and stemness that may not be readily captured by single-omics approaches.

From an evolutionary perspective, the gastritis-carcinoma sequence may represent a dynamic process of cellular and molecular reprogramming rather than a series of isolated pathological stages. Early inflammatory and metabolic alterations in CG may contribute to epithelial plasticity and lineage reprogramming during progression to SPEM and IM. During this process, clonal expansion, epigenetic remodeling, and microenvironmental selection collectively influence disease trajectories, while spatial heterogeneity may facilitate the emergence of high-risk cellular subpopulations. Importantly, this progression is unlikely to follow a strictly linear pattern and instead involves continuous interactions among host cells, microbiota, and the immune microenvironment.

Recent advances in spatial and single-cell multi-omics, together with multi-kingdom microbiome profiling, are beginning to improve understanding of the spatiotemporal evolution of gastric lesions in human tissues. These approaches provide insights into disease mechanisms and may support the identification of transition states, candidate biomarkers, and risk stratification strategies across the Correa cascade, although further multicenter validation and prospective assessment are still required before clinical translation.

### Critical appraisal of current omics studies and integration strategies

Despite the rapid expansion of omics-based studies in CG and GPL, several important limitations should be considered from both methodological and integrative perspectives.

First, many scRNA-seq studies are limited by relatively small sample sizes and substantial patient heterogeneity, which may reduce the robustness and generalizability of identified cellular subpopulations and transcriptional programs. In addition, batch effects and differences in preprocessing pipelines across studies introduce technical variability, complicating cross-cohort comparisons and integrative analyses. Second, a substantial proportion of current findings remains associative, with insufficient functional validation to establish causal relationships. For example, although regulators such as ITGB2 and STAT3-related pathways have been implicated across multiple stages of the gastritis-carcinoma sequence, mechanistic validation *in vivo* or in organoid systems remains limited.

Third, inconsistencies across studies and omics layers are increasingly evident. Divergent microbial signatures, heterogeneous gene expression patterns, and context-dependent metabolic alterations have been reported in CNAG, CAG, and IM, reflecting both biological complexity and technical variability. However, these discrepancies are rarely systematically addressed. In addition, many current multi-omics integration strategies remain largely descriptive, focusing on parallel presentation of transcriptomic, proteomic, or metabolomic findings rather than identifying convergent regulatory pathways or functionally connected networks. Several domain-specific limitations also remain. Most microbiome studies rely predominantly on 16S rRNA-seq, which mainly captures bacterial communities and provides limited taxonomic and functional resolution. Emerging evidence suggests that non-bacterial components, including the mycobiome and virome, may also contribute to gastric carcinogenesis. Fungal dysbiosis has been reported in GC tissues, characterized by altered diversity and enrichment of taxa such as Solicoccozyma and Malassezia, which are associated with tumor progression and microenvironmental remodeling.^149^^,^^150^ Fungal communities may also participate in immune modulation and inflammatory responses, although causal relationships remain unclear.^154^

In contrast, the gastric virome remains poorly characterized. Current knowledge is largely limited to oncogenic viruses such as Epstein-Barr virus, while broader viral communities and their interactions with bacterial and fungal components remain insufficiently explored.^125^ Advances in shotgun metagenomic sequencing now enable more comprehensive profiling of bacterial, fungal, and viral communities, supporting multi-kingdom microbiome analyses.^155^ However, integrative studies across the gastritis-carcinoma sequence remain limited. Another important limitation is the risk of false-positive findings arising from large-scale multiple hypothesis testing. In some studies, insufficient reporting or inconsistent application of multiple-testing correction methods, such as false discovery rate (FDR) control, may affect the reliability and comparability of results across studies.

Therefore, future research should prioritize rigorous cross-validation, standardized analytical frameworks, and function-oriented multi-omics integration to distinguish reproducible molecular drivers from context-specific findings. Strengthening causal inference through experimental models and focusing on pathway-level convergence across omics layers will be important for advancing mechanistic understanding and supporting future clinical translation.

### Current challenges and innovative solutions

Despite substantial advances in omics technologies for CG and GPL research, several major challenges remain.

First, comprehensive data integration remains limited because no single omics layer fully captures lesion dynamics, while cross-platform integration is often hindered by batch effects and data heterogeneity. Second, causal inference remains insufficient, as most studies are observational and cannot clearly distinguish disease drivers from downstream consequences. For example, microbial alterations such as DCA-associated Lactobacillus proliferation with STAT3/KLF5 activation^123^ or Peptostreptococcus enrichment following *H. pylori* eradication^126^ have not been fully validated mechanistically. Similarly, key regulatory axes identified across omics layers, including the ITGB2-CXCL1-CXCR2 axis,^31^ are still supported mainly by correlative evidence with limited functional validation. Third, clinical translation remains constrained by the lack of large-scale multicenter validation, while invasive sampling procedures limit practical applicability. Inter-individual variability further complicates the identification of robust and generalizable biomarkers. Fourth, ethical and practical limitations restrict longitudinal sampling of GPL, resulting in a shortage of prospective datasets for dynamic disease modeling and validation.

To address these challenges, future studies should prioritize integrative frameworks with improved causal inference, supported by organoid models, longitudinal cohorts, and large-scale prospective validation. Standardized analytical workflows and computational tools will also be essential for improving reproducibility and comparability across studies. Table 7 summarizes representative analytical workflows and computational tools for multi-omics integration and biomarker discovery in GPL research, including preprocessing, differential analysis, functional interpretation, network analysis, spatial deconvolution, and model validation. Because study design, disease stratification, and data availability vary substantially across cohorts, analytical pipelines should be transparently reported and externally validated before derived signatures are considered clinically robust.

**Table 7:** Analytical workflows and representative tools for multi-omics data integration and biomarker discovery in gastric precancerous lesions

Omics layer	Analytical stage	Key approaches	Representative tools	Biological insights/outputs
Bulk transcriptomics	Data preprocessing	Quality control, normalization	FastQC, DESeq2	Reliable gene expression matrix
	Differential analysis	DEG identification	DESeq2, edgeR, limma	Candidate genes (CAG/IM)
	Functional interpretation	Pathway enrichment, GSEA	clusterProfiler, GSEA	Dysregulated pathways
	Network analysis	Co-expression networks	WGCNA	Hub genes, regulatory modules
	Translational validation	Signature construction	LASSO, Cox regression	Candidate diagnostic/prognostic signatures requiring external validation
Single-cell transcriptomics	Data processing	Filtering, normalization	Seurat, Scanpy	High-quality single-cell data
	Dimensional reduction	PCA, UMAP/t-SNE	Seurat	Cell clustering
	Cell annotation	Marker-based annotation	SingleR, CellMarker	Cell-type identification
	Cell–cell interaction	Ligand–receptor analysis	CellChat, NicheNet	Microenvironment interactions
	Trajectory inference	Pseudotime analysis	Monocle, Slingshot, CytoTRACE	Lineage evolution (e.g., SPEM→IM)
Spatial transcriptomics	Spatial mapping	Spatial clustering	STUtility, Giotto, Space Ranger	Tissue architecture
	Integration with scRNA	Deconvolution	SPOTlight, Seurat, RCTD	Spatial cell composition
	Spatial interaction	Niche analysis	Squidpy	Microregional heterogeneity
Proteomics	Protein quantification	Label-free/TMT	MaxQuant, Perseus	Differential proteins
	Functional analysis	Pathway enrichment	IPA, DAVID	Functional protein networks
	Network analysis	Protein–Protein Interaction	STRING, Cytoscape	Key hubs and interaction modules
Metabolomics	Metabolite profiling	Untargeted/targeted analysis	XCMS, MetaboAnalyst	Metabolic signatures
	Pathway analysis	KEGG mapping	MetaboAnalyst	Metabolic pathways
Microbiomics	Taxonomic profiling	16S/metagenomics	QIIME2, MetaPhlAn, Kraken2	Microbial composition
	Functional profiling	Pathway inference	HUMAnN	Microbial functions
Multi-omics integration	Data integration	Multi-layer integration	MOFA, iCluster, DIABLO	Cross-omics patterns
	Machine learning	Feature selection, modeling	Random Forest, SVM, deep learning	Biomarker prioritization
	Network modeling	Multi-layer networks	Cytoscape	Mechanistic pathways

Abbreviations: GPL, gastric precancerous lesions; CAG, chronic atrophic gastritis; IM, intestinal metaplasia; DEG, differentially expressed genes; GSEA, gene set enrichment analysis; WGCNA, weighted gene co-expression network analysis; LASSO, least absolute shrinkage and selection operator; PCA, principal component analysis; UMAP, uniform manifold approximation and projection; t-SNE, t-distributed stochastic neighbor embedding; SPEM, spasmolytic polypeptide-expressing metaplasia; RCTD, robust cell type decomposition; TMT, tandem mass tag; IPA, Ingenuity Pathway Analysis; DAVID, Database for Annotation, Visualization and Integrated Discovery; KEGG, Kyoto Encyclopedia of Genes and Genomes; 16S, 16S ribosomal RNA sequencing; MOFA, multi-omics factor analysis; DIABLO, Data Integration Analysis for Biomarker discovery using Latent Components; SVM, support vector machine.

Machine learning-based multi-omics integration has also emerged as a potential strategy for exploratory pattern recognition and risk stratification across disease stages such as CAG, IM, and SPEM. Computational approaches, including unsupervised clustering, network-based integration, and deep learning, can integrate heterogeneous datasets derived from transcriptomics, proteomics, metabolomics, and microbiome profiling. At present, these methods are best viewed as tools for generating testable molecular subtypes, reconstructing disease trajectories, and characterizing regulatory networks associated with the inflammation-to-metaplasia transition.

Such integrative models may improve disease classification and risk stratification by identifying stage-specific molecular signatures and shared regulatory pathways across CAG, IM, and SPEM. However, their application remains limited by data heterogeneity, potential overfitting, limited interpretability, and insufficient external validation, which continue to restrict robustness and clinical applicability.

### Complementary perspectives from intervention-oriented multi-omics studies

Intervention-oriented multi-omics studies provide complementary mechanistic perspectives but should not dominate the interpretation of the gastritis-to-carcinoma sequence. Existing studies have linked selected herbal or natural-product interventions to inflammatory signaling, microbiota-bile acid interactions, and ferroptosis-related pathways.^25^^,^^31^^,^^71^^,^^119^ These observations may help generate mechanistic hypotheses, but most are derived from animal models, network-based prediction, or small clinical cohorts and therefore remain secondary to evidence from human multi-omics and translational studies.

These findings suggest that intervention-related omics studies may inform pathway-level hypotheses involving inflammatory responses, metabolic remodeling, and microbiota-associated alterations. However, the current evidence base remains heterogeneous and is generally limited by relatively low levels of clinical evidence. Many studies are based on animal models, *in vitro* experiments, computational target prediction, or small-scale clinical cohorts, often lacking rigorous randomization, blinding, and appropriate control groups. Although some clinical studies have reported beneficial effects, limited sample sizes and short follow-up durations restrict reproducibility and generalizability. Therefore, these studies should be presented as complementary mechanistic evidence rather than as established clinical guidance.

In addition, several potential sources of bias should be considered. Publication bias may contribute to overrepresentation of positive findings, while variability in herbal formulations, dosage regimens, and syndrome differentiation introduces substantial heterogeneity across studies. The lack of standardized protocols and unified outcome measures further limits cross-study comparisons and meta-analytic evaluation. Moreover, although multi-omics analyses provide valuable mechanistic insights, most reported associations remain correlative and require further validation in well-controlled experimental systems.

Therefore, future intervention studies should prioritize high-quality, large-scale randomized controlled trials with standardized protocols, longer follow-up periods, and predefined molecular endpoints. Integrating multi-omics analyses into such trials may help strengthen mechanistic interpretation while ensuring that translational claims remain grounded in reproducible clinical evidence.

### Future directions: Advancing the transition from mechanistic research to clinical application

**Figure 2:**
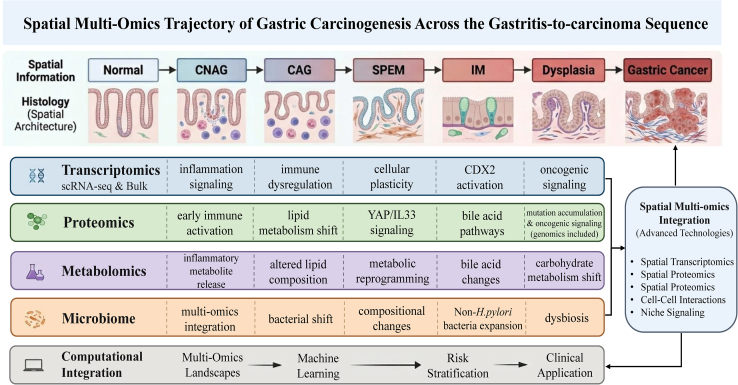
Conceptual spatial multi-omics trajectory of gastric carcinogenesis across the gastritis-to-carcinoma sequence The framework summarizes stage-associated transcriptomic, proteomic, metabolomic, microbiome, and computational features from normal mucosa through CNAG, CAG, SPEM, IM, dysplasia, and GC. The depicted trajectories are intended to guide hypothesis generation and require validation in independent human cohorts.

To further integrate current knowledge and outline future research priorities, a spatial multi-omics trajectory framework of gastric carcinogenesis is proposed. Figure 2 illustrates the dynamic interactions between multi-layer molecular alterations and spatial microenvironmental remodeling across the gastritis-to-carcinoma sequence, highlighting the evolutionary trajectory of disease progression revealed by spatial multi-omics analyses.

Future research should prioritize continued development of omics technologies, particularly spatial multi-omics, to better characterize microregional heterogeneity and spatial microenvironmental dynamics in GPL.^1^^,^^2^ Integrative analyses combining transcriptomics, proteomics, and metabolomics with computational approaches may improve the interpretation of gene-environment interactions and facilitate the identification of biomarkers with potential clinical relevance.^3^^,^^4^ However, such biomarkers should be advanced through staged validation, including independent cohorts, standardized assays, and prospective assessment. In parallel, organoid models recapitulating the “inflammation-to-cancer” sequence, together with robust validation platforms, may provide useful systems for functional validation of molecular findings and preclinical therapeutic evaluation.^5^ Establishment of comprehensive human multi-omics biobanks and molecular subtype classification systems may further support precision-oriented therapeutic strategies.^6^ In addition, integrating liquid biopsy approaches with spatiotemporal omics analyses may improve the detection of early disease-associated alterations.^7^ Collectively, these strategies may enhance understanding of spatial disease dynamics and support more individualized management approaches, although clinical implementation remains dependent on large-scale validation.

Recent advances in spatial multi-omics and artificial intelligence-assisted analysis have further expanded the analytical framework for investigating the gastritis-carcinoma sequence.^1^^,^^3^ Spatial multi-omics integrates transcriptomic, proteomic, and metabolomic information within intact tissue architecture, enabling higher-resolution characterization of lesion heterogeneity compared with bulk or conventional single-cell analyses alone.^2^ Emerging evidence from human tissue-based studies suggests that *H. pylori*-associated lesions exhibit marked spatial heterogeneity in microbial distribution, immune infiltration, and epithelial transformation.^8^

**Figure 3:**
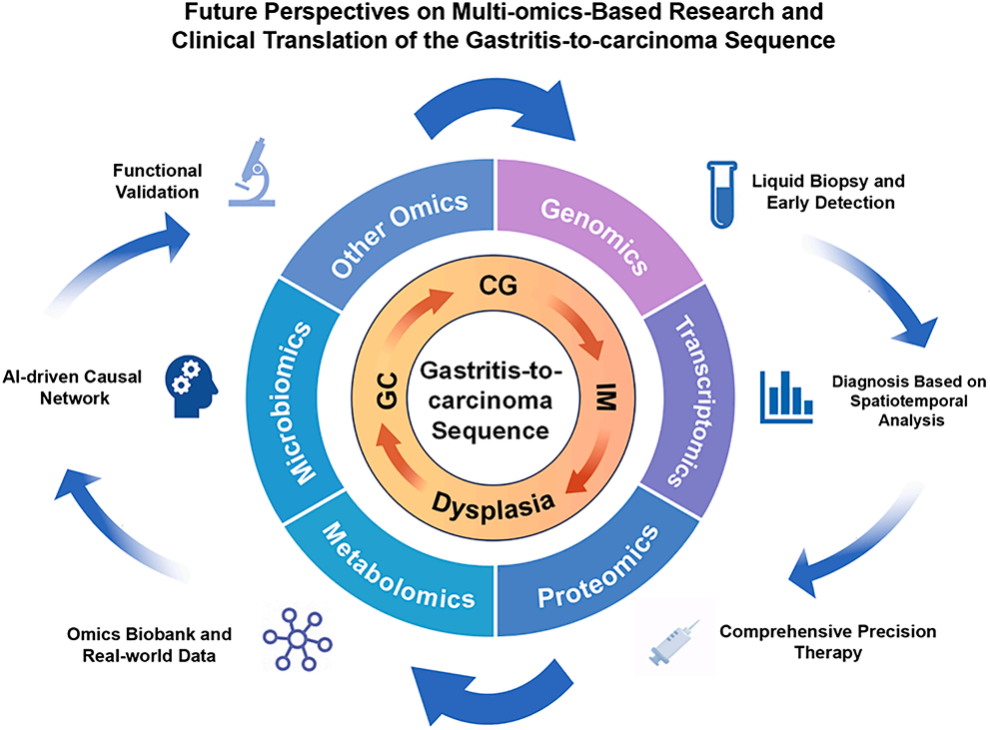
Future perspectives for multi-omics-based research and translational validation in the gastritis-to-carcinoma sequence The figure highlights biobanks, real-world data, functional validation, liquid biopsy, spatiotemporal analysis, and AI-assisted computational modeling as complementary research directions. AI-assisted and machine-learning outputs should be considered exploratory until validated in standardized, prospective, and externally replicated studies.

In parallel, machine learning and deep learning approaches are increasingly being explored for multi-omics data integration, biomarker discovery, and risk stratification.^3^^,^^4^ By integrating heterogeneous molecular datasets, these computational approaches may help identify predictive signatures associated with precancerous lesions. However, current applications remain exploratory and are limited by data heterogeneity, relatively small sample sizes, potential overfitting, limited interpretability, and insufficient external validation. Future efforts should therefore focus on standardized analytical frameworks, transparent model reporting, and validation in independent and prospective cohorts before these models are considered clinically actionable. Figure 3 illustrates a data-driven framework for translational research integrating biobanks and computational analyses for biomarker discovery and pathogenic mechanism exploration.

Data-driven strategies may also support exploratory evaluation of complementary intervention hypotheses by linking curated intervention databases with GPL-related multi-omics datasets.^9^ Such analyses may help prioritize molecular targets for experimental testing, but they should be clearly distinguished from established clinical evidence and require substantial validation before informing prevention or treatment strategies.

## Conclusion

In summary, the integration of multi-omics—including genomics, transcriptomics, proteomics, metabolomics, microbiomics, and epigenomics—has substantially advanced the understanding of CG and GPL progression and facilitated the identification of key biomarkers and regulatory networks. Current evidence highlights the coordinated involvement of host-microbiome interactions, metabolic reprogramming, immune dysregulation, and epigenetic alterations in disease progression. Moreover, the gastritis-carcinoma sequence appears to represent a dynamic evolutionary process characterized by clonal evolution, epithelial plasticity, and microenvironmental remodeling. Despite these advances, important challenges remain, particularly regarding cross-omics data integration, causal validation, and clinical translation. Future studies should prioritize standardized analytical frameworks, large-scale multicenter validation cohorts, and function-oriented experimental models to improve the robustness and reproducibility of current findings. Emerging spatial multi-omics and computational approaches may further refine molecular characterization and risk stratification across disease stages; however, their clinical applicability still requires rigorous validation. Collectively, these efforts may contribute to more precise and evidence-based strategies for the early detection and management of CG and GPL.

## Acknowledgments

The authors acknowledge that AI-assisted tools (e.g., ChatGPT) were used for language editing to improve clarity and readability. All scientific content, analyses, and interpretations remain the sole responsibility of the authors. Some icons used in the figures were obtained from free resources (e.g., Scientific Drawings, Servier Medical Art) and were used in compliance with their respective license terms.

This work was supported by the following grants: the National Key Research and Development Program on Cancer, Cardiovascular and Cerebrovascular Diseases, Respiratory and Metabolic Diseases Prevention and Treatment (2024ZD0521000); the National Science and Technology Innovation 2030 Program: Four Major Chronic Disease Special Projects-University-Enterprise Joint Collaboration (DZ-09-202510-01); the 10.13039/501100010618Guangzhou University of Chinese Medicine “Double First-Class” Discipline High-Quality Development “Building Peaks and Sharpening Tips” Action Plan (grant no. GZY2025ZJ08); the Youth Development Fund Project of Guangdong Traditional Medical Association (GDTMAZX-202509004); the Guangzhou University of Traditional Chinese Medicine “Announcing the Winners” Postgraduate Innovation Project (D-2-10); the Special Funds for the Cultivation of Guangdong College Students’ Scientific and Technological Innovation (“Climbing Program” Special Funds) (pdjh2024a098); and the Chongqing Science and Health Joint Medical Research Project (2026MSXM151); Scientific Research Project of Chongqing Beibei District (2025shsyfz-60).

## Author contributions

Writing – original draft, J.O. and J.L.; conceptualization, Z.X. and H.Q.; investigation, J.O., J.L., Y.C., Y.C., J.L., J.F., J.Li, Jd.L., H.Q., and Z.X.; writing – review and editing, J.O., J.L., Y.C., Y.C., J.L., J.F., J.L., Jd.L., H.Q., and Z.X.; visualization, J.O. and J.L.

## Declaration of interests

The authors declare no conflict of interest.
